# Proteomic profiling of lung adenocarcinoma indicates heightened DNA repair, antioxidant mechanisms and identifies LASP1 as a potential negative predictor of survival

**DOI:** 10.1186/s12014-016-9132-y

**Published:** 2016-10-27

**Authors:** Johannes F. Fahrmann, Dmitry Grapov, Brett S. Phinney, Carol Stroble, Brian C. DeFelice, William Rom, David R. Gandara, Yanhong Zhang, Oliver Fiehn, Harvey Pass, Suzanne Miyamoto

**Affiliations:** 1 University of California, Davis Genome Center, Davis, CA USA; 2 CDS Creative Data Solutions, Ballwin, MO USA; 3 Genome Center Proteomics Core Facility, University of California, Davis, Davis, CA USA; 4 Division of Hematology and Oncology, Department of Internal Medicine, University of California, Davis Medical Center, 4501 X Street, Suite 3016, Sacramento, CA 95817 USA; 5 Division of Pulmonary, Critical Care, and Sleep, NYU School of Medicine, New York, NY USA; 6 Department of Pathology and Laboratory Medicine, University of California, Davis Medical Center, Sacramento, CA USA; 7 Department of Biochemistry, Faculty of Sciences, King Abdulaziz University, Jeddah, 21589 Saudi Arabia; 8 Division of Thoracic Surgery, Department of Cardiothoracic Surgery, Langone Medical Center, New York University, New York City, NY USA; 9 Department of Clinical Cancer Prevention, University of Texas MD Anderson Cancer Center, Houston, TX USA

**Keywords:** Lung adenocarcinoma, Proteomics, Biomarker

## Abstract

**Background:**

Lung cancer is the leading cause of cancer mortality in the United States. Non-small cell lung cancer accounts for 85% of all lung cancers for which adenocarcinoma is the most common histological type. Management of lung cancer is hindered by high false-positive rates due to difficulty resolving between benign and malignant tumors. Better molecular analysis comparing malignant and non-malignant tissues will provide important evidence of the underlying biology contributing to tumorigenesis.

**Methods:**

We utilized a proteomics approach to analyze 38 malignant and non-malignant paired tissue samples obtained from current or former smokers with early stage (Stage IA/IB) lung adenocarcinoma. Statistical mixed effects modeling and orthogonal partial least squares discriminant analysis were used to identify key cancer-associated perturbations in the adenocarcinoma proteome. Identified proteins were subsequently assessed against clinicopathological variables.

**Results:**

Top cancer-associated protein alterations were characterized by: (1) elevations in APEX1, HYOU1 and PDIA4, indicative of increased DNA repair machinery and heightened anti-oxidant defense mechanisms; (2) increased LRPPRC, STOML2, COPG1 and EPRS, suggesting altered tumor metabolism and inflammation; (3) reductions in SPTB, SPTA1 and ANK1 implying dysregulation of membrane integrity; and (4) decreased SLCA41 suggesting altered pH regulation. Increased protein levels of HYOU1, EPRS and LASP1 in NSCLC adenocarcinoma was independently validated by tissue microarray immunohistochemistry. Immunohistochemistry for HYOU1 and EPRS indicated AUCs of 0.952 and 0.841, respectively, for classifying tissue as malignant. Increased LASP1 correlated with poor overall survival (HR 3.66 per unit increase; CI 1.37–9.78; p = 0.01).

**Conclusion:**

These results reveal distinct proteomic changes associated with early stage lung adenocarcinoma that may be useful prognostic indicators and therapeutic targets.

**Electronic supplementary material:**

The online version of this article (doi:10.1186/s12014-016-9132-y) contains supplementary material, which is available to authorized users.

## Background

Lung cancer is a leading cause of cancer mortality in both men and women in the United States [1–3]. Non-small cell lung cancer (NSCLC) accounts for 85% of all lung cancer cases for which NSCLC adenocarcinoma is the most common histological type [4]. While use of low dose computerized tomography (LDCT) for screening of persons at high risk for lung cancer can reduce cancer mortality, it is plagued by high false positive rates (96%) [5] because it is unable to adequately distinguish indolent (benign) solid pulmonary nodules (SPNs) from malignant SPNs. Increased knowledge of the molecular perturbations caused by tumorigenesis is needed to better understand the underlying biology, as well as potentially assisting with diagnosis, prognosis and identification of additional treatment targets.

Improved ‘Omic’ based analytical methods (e.g. genomics, transcriptomics, proteomics and metabolomics) gives us greater ability to monitor all biochemical processes associated with tumorigenesis with increasingly smaller amounts of difficult-to-obtain clinical specimens. Proteomics is particularly well suited to identify the underlying biology of lung cancer. Protein expression is the ultimate product of gene expression and is controlled through transcriptional, translational and post-translational regulations, all of which are highly dependent on cellular signaling [6]. Unlike genomics (DNA and RNA), proteomic analysis is more complex due to the presence of sequence variations, isoforms, and post-translational modifications, yielding multiple protein isoforms of the same gene Proteomics can uncover powerful links between gene function and tumorigenesis, help discover clinically useful diagnostic and prognostic biomarkers for early stage NSCLC adenocarcinoma [7–9]. In the current study, a shotgun tandem liquid chromatography mass spectrometry (LC–MS/MS) approach was used to characterize proteomic differences between 38 matched malignant and non-malignant lung tissue pairs obtained from current or former smokers with early stage (IA-IB) NSLCL adenocarcinoma. Statistical analysis and multivariate modeling were used to identify the top 10% of all measured protein changes that best distinguished adenocarcinoma from control tissues. Identified proteins were additionally evaluated against clinicopathological variables. Three top candidate proteins, EPRS, HYOU1 and LASP1 from the discovery study were independently validated with a tissue microarray containing 40 pairs of malignant and non-malignant tissues from patients with early stage NSCLC adenocarcinoma. We hypothesize that identification of cancer induced cellular and tissue level protein changes will provide candidate tissue-specific prognostic markers for early stage adenocarcinoma that may eventually be used to better distinguish adenocarcinoma from benign tissues, help identify potential therapeutic targets for treatment of lung cancer and, importantly, improve our understanding of the mechanism(s) leading to lung cancer.

## Methods

### Sample acquisition

De-identified malignant and adjacent non-malignant lung tissue samples were harvested in the operating room from patients having resection or lobectomy for non-small cell lung cancer, none of whom received preoperative treatment. The matching control lung tissue was always taken from areas 8–10 cm removed from the cancer bed. All procedures were approved by institutional IRB protocols (NYU) with patient consent. Tissues were immediately frozen in liquid nitrogen and stored at −80 °C. Specimens were annotated for age, gender, race, diagnosis (including stage), smoking status and pack-years. Criteria used to select patient cases were: (a) current or former smokers; (b) diagnosis of NSCLC adenocarcinoma; (c) early stage IA or IB; and (d) understood and signed the IRB consent form. Of the 38 samples analyzed in the study, 14 (36.8%) were from patients that had recurrent cancer within 5 years. The inclusion of these 14 subjects was to identify potential prognostic biomarkers of early stage adenocarcinoma.

### Proteomic analysis

#### Preparation of samples for proteomic analysis

Preparation of tissues and *N*-glycan release followed by ethanol precipitation of tissue proteins has been previously described in Ruhaak et al. [10].

#### Trypsin digestion of samples

Protein pellets were solubilized in 100 µL of AMBIC (6 M urea, 50 mM ammonium bicarbonate) with dithiothreitol added (final concentration 5 mM) and incubated at 37 °C for 30 min. Iodoacetamide (IAA) was added (final concentration 15 mM), incubated for 30 min (RT) and DTT added to quench the IAA reaction. Lys-C/trypsin (Promega) was next added (1:25 enzyme:protein ratio), samples incubated (4 h at 37 °C) after which the urea concentration was lowered (<1 M) and samples further digested (overnight, 37 °C). Samples were desalted (C18 Macro Spin columns, Nest Group) and lyophilized.

#### LC–MS/MS analysis

Reconstituted samples were randomized into 8 blocks of 4 normal and 4 tumor samples. Triplicate LC–MS/MS analyses were acquired for each sample. LC separation was done on a Waters Nano Acquity UHPLC (Waters Corporation) with a Proxeon nanospray source. Mass spectra was collected on an Orbitrap Q Exactive Plus mass spectrometer (Thermo Fisher Scientific) in a data-dependent mode with one MS precursor scan followed by 15 MS/MS scans as previously described [11]. Detailed information on instrument parameters and mass spectra collection is provided in the Additional file 1: Supplemental materials and methods (1a). The mass spectrometry proteomics data have been deposited to the ProteomeXchange Consortium [12] via the PRIDE partner repository with the dataset identifier PXD002612.

#### Protein identification

Protein identification was performed similarly to Zhang et al. [13]. Detailed information can be found in Additional file 1: Supplemental materials and methods (1b).

### Immunohistochemistry of lung cancer LC003 TMA

#### Preparation of LC003 TMA

A tissue microarray (LC003) was prepared from FFPE tissue blocks containing non-malignant and malignant (tumor) tissues obtained from 40 patients diagnosed with early stage (Stage I and II) NSCLC adenocarcinoma after patient consent using an IRB approved protocol (IRB 293828, UC Davis Cancer Center Biorepository). Three 0.6 mm (diameter) by 0.4 mm (length) cylindrical cores of tumor and adjacent non-malignant (control) lung tissues were collected from each case and placed in the same block (Quick-Ray Manual Tissue Microarrayer). One TMA section was stained with H&E (hematoxylin and eosin) to confirm the presence of tumor and normal lung tissue. Clinical data (gender, age, smoking history, diagnosis and stage) information was provided after analysis.

#### Immunohistochemistry of LC003 TMA

Immunohistochemical staining was performed on 4-μm FFPE tissue sections from the LC003 TMA for three different protein targets, HYOU1, EPRS and LASP1. Details of the IHC procedure are provided in Additional file 1: Supplemental materials and methods (1c).

Immunostained slides were scored by a pathologist (YZ) blinded to clinicopathologic information. Staining for HYOU, EPRS and LASP1 was semi-quantified assessed using both intensity and percentage of positive cells. Staining intensity was graded as 0 = negative (no cells stained); 1 = weak; 2 = moderate; 3 = strong. The H score was calculated by multiplying the percentage of positive cells demonstrating each density (score 0 to 3) and adding the results. The average H score of 3 cores in a case was calculated. High expression was defined as H score 251–300; medium expression was H score 151–250; low expression was H score less than 150.

### Statistical analysis

Detailed information on statistical analyses are provided in Additional file 1: Supplemental Materials and methods (1d). A conservative approach was used to overview data quality metrics in order to identify the most robust proteomic measurements for downstream statistical and multivariate analyses [11]. A total of 799 high quality protein measurements (spectral counts) based on absence of abundant missing zeros, replicate precision, protein sequence coverage and annotation to known genes were selected for further statistical and multivariate analyses.

Statistical analyses were conducted on mean normalized count data. Statistical tests were conducted using generalized negative binomial mixed effects regression with the patients as the random term [14]. Significantly altered proteins were identified based on the comparison of the full model (Chi squared test) including age + gender + packs + tumor/control to a reduced model excluding the tumor/normal labels. Model p-values were adjusted for multiple hypotheses tested [15].

Orthogonal partial least squares discriminant analysis (O-PLS-DA) multivariate models were used to identify the top 10% of all protein discriminants between tumor and control tissues as previously described [16].

A Gaussian graphical model protein–protein empirical network was calculated for O-PLS-DA selected top discriminants (n = 16) as previously described [16].

All subjects were included in the survival analysis with the exception of six subjects who did not die of lung cancer 23–2262 days from their surgery, and who, before their death, never had recurrent lung cancer. Kaplan–Meier survival curves were generated using Prism v5.0 (GraphPad Software, Inc). Significance was determined using log-rank Mantel-Cox test. Cox proportional hazard models were carried out in R statistical software.

### *LASP1* transcriptomic data

*LASP1* transcriptomic data was obtained from the Okayama NSCLC study [17] using the Oncomine Database [18]. The study set was chosen due its specific focus on early stage NSCLC adenocarcinoma and availability of clinicopathological variables. Only Stage I (IA/IB) adenocarcinoma subjects were considered. Subject characteristics are provided in Additional file 1: Table S5. *LASP1* values were log_2_-median centered normalized. Cox proportional hazard models were used to evaluate the association between *LASP1* mRNA expression and overall survival.

## Results

Paired tissue samples were obtained from 38 patients with adenocarcinoma histology (Table 1). The majority of subjects were white female former smokers. The average age was 70 with a mean of 33 packs per year; subjects were diagnosed with stage IA or IB adenocarcinoma. Of the 38 patients, 14 (36.8%) progressed.

**Table 1:** Patient characteristics

Variable	Lung cancer patients
Total sample size, N	38
Gender, N (males/female)	14/24
Age, mean ± SD (min, max)	70 ± 9 (56, 91)
Packs per year, mean ± SD (min, max)	33 ± 25 (0, 100)
Current smoker, N (%)	6 (15.79%)
Stage	
Stage IA, N (%)	21 (55.3%)
Stage IB, N (%)	17 (44.7%)
Developed lymphovascular invasion, N (%)	6 (15.8%)
Developed pleural invasion, N (%)	13 (34.2%)
Progressed, N (%)	14 (36.84%)
Local–regional, N	4
Second primary, N	3
Distal, N	7

**Fig. 1:**
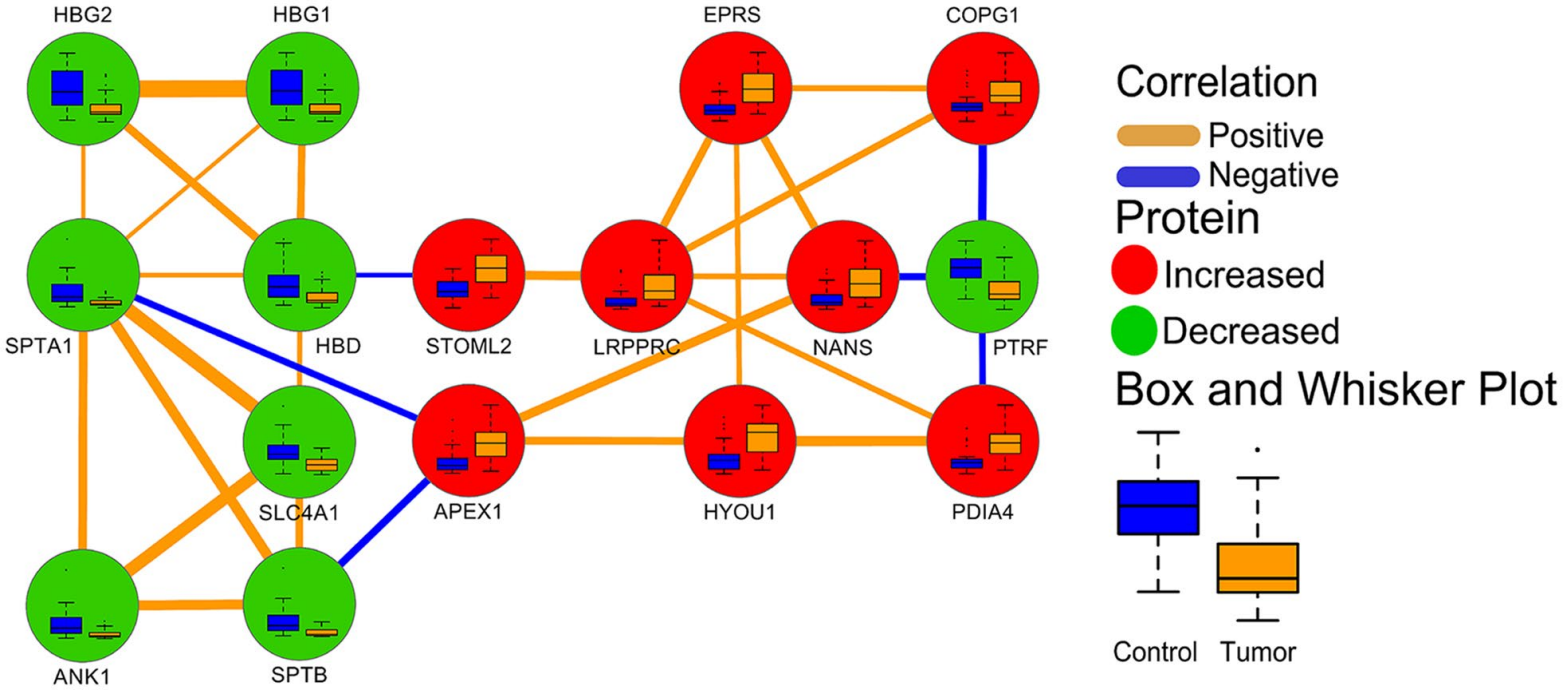
Gaussian graphical model empirical network for O-PLS-DA selected top 10% discriminants between normal and tumor tissues. *Edge color* and width denote the direction and magnitude of partial correlations (pFDR ≤ 0.05). *Node color* displays the direction of the change in tumor relative to non-malignant tissue (*green*, decrease; *red*, increase; pFDR ≤ 0.05). *Node inset box* and *whisker plots* summarize differences in spectral measurements between tumor and non-malignant tissue

Proteomic profiling was performed on matched malignant and control tissue and yielded a total of 10,712 protein groups (see “Methods”). A conservative filter criteria approach was used to select 799 of the most robust proteomic measurements for further statistical analyses (Additional file 2: Table S1). Generalized negative binomial mixed effects regression models were used to identify 436 differentially expressed proteins in lung adenocarcinoma relative to control tissue, of which 367 remained significantly different following FDR adjustment (Additional file 1: Table S2). O-PLS-DA multivariate classification modeling was used to select the top 10% multivariate discriminants between tumor and control tissues (Table 2). Monte Carlo cross-validation and permutation testing were used to validate the models predictive performance for classification of cancer vs. control tissues (Additional file 1: Table S3). The top 10% discriminants between tumor and control tissue consisted of 16 proteins with 8 being significantly higher in tumor tissue relative to control (Table 2). A Gaussian graphical model network was calculated to identify conditionally independent relationships (partial correlation, pFDR ≤ 0.05) between the top discriminatory proteins for adenocarcinoma, the relationships between which were finally expressed as non-parametric Spearman’s rank correlations (FDRp < 0.05) (Fig. 1).

**Table 2:** Top 10% discriminants of adenocarcinoma

Gene ID	Gene name	Control (mean ± SD)^a^	Tumor (mean ± SD)^a^	Fold change^b^	p value^c^	Ratio^d^	Percent (%)^e^	Rank^f^
SPTB	Spectrin, beta, erythrocytic	10.7 ± 9.3	2.82 ± 2.6	0.3	<0.0000	(7/38)	18	1
SLC4A1	Solute carrier family 4 (anion exchanger), member 1 (Diego blood group)	8.75 ± 5.2	3.8 ± 2.7	0.4	<0.0000	(9/38)	24	2
SPTA1	Spectrin, alpha, erythrocytic 1	13.3 ± 12	4.28 ± 3.4	0.3	<0.0000	(8/38)	21	3
ANK1	Ankyrin 1, erythrocytic	6.28 ± 5.7	1.84 ± 1.8	0.3	0.0001	(8/38)	21	4
EPRS	Glutamyl-prolyl-tRNA synthetase	1.24 ± 0.87	3.15 ± 1.7	2.5	<0.0000	(30/38)	79	5
HBG1	Hemoglobin, gamma A	16.9 ± 9.7	7.44 ± 6.2	0.4	<0.0000	(9/38)	24	6
HBG2	Hemoglobin, gamma G	17 ± 9.7	7.43 ± 6.2	0.4	<0.0000	(9/38)	24	7
COPG1	Coatomer protein complex, subunit gamma 1	1.56 ± 1	2.67 ± 1.4	1.7	0.0036	(29/38)	76	8
HYOU1	Hypoxia up-regulated 1	2.12 ± 1.9	4.74 ± 2.3	2.2	<0.0000	(31/38)	82	9
PTRF	Polymerase I and transcript release factor	7.35 ± 2.8	3.81 ± 2.8	0.5	0.0001	(4/38)	11	10
PDIA4	Protein disulfide isomerase family A, member 4	2.69 ± 2.2	6.23 ± 3.4	2.3	0.0003	(31/38)	82	11
APEX1	APEX nuclease (multifunctional DNA repair enzyme) 1	0.778 ± 0.83	2.05 ± 1.1	2.6	<0.0000	(32/38)	84	12
STOML2	Stomatin (EPB72)-like 2	0.675 ± 0.39	1.38 ± 0.57	2	0.0084	(32/38)	84	13
LRPPRC	Leucine-rich pentatricopeptide repeat containing	1.31 ± 1.4	3.52 ± 2.5	2.7	0.0001	(29/38)	76	14
NANS	*N*-acetylneuraminic acid synthase	0.61 ± 0.55	1.64 ± 1.1	2.7	0.0002	(31/38)	82	15
HBD	Hemoglobin, delta	47.3 ± 32	21.7 ± 18	0.5	0.0001	(8/38)	21	16

^a^Values represent covariate adjusted spectral counts

^b^Ratio of means relative to control

^c^False discovery rate adjusted mixed effects model *p* value

^d^Ratio represents the number of tumor samples which indicated a higher abundance relative to matched control tissue

^e^Percent (%) represents number of cases were the respective protein was increased in tumor relative to control

^f^Importance of metabolic change based on O-PLS-DA model loading

### NSCLC adenocarcinoma is characterized by alterations in DNA repair mechanisms, antioxidant defense capacity, altered membrane integrity and metabolism

SPTB was determined to be the single most discriminatory protein of adenocarcinoma displaying a 70% reduction in tumor tissue relative to control tissue (Table 2). The adenocarcinoma-dependent reduction in SPTB was also consistently observed in 82% of subjects. Tumor-associated reductions in SPTB were associated with similar reductions in SPTA1 (70%), SLC4A1 (60%), and ANK1 (70%) in tumor tissue when compared to control tissue (Fig. 1; Table 2). The reduction in SPTA1 was also directly correlated with similar reductions in the hemoglobin subunits HBD, HBG1 and HBG2, which were also found to be directly correlated among each other (Fig. 1). Reductions in SPTA1 and SPTB were indirectly associated with APEX1, which showed a 2.7-fold increase in adenocarcinoma compared to non-malignant tissue and was consistently elevated in 82% of subjects (Fig. 1; Table 2). The increase in APEX1 was correlated with similar increases in HYOU1 and NANS, which were also increased 2.2- and 2.7-fold in malignant compared to non-malignant tissue (Fig. 1; Table 2). HYOU1 and NANS were both positively correlated with adenocarcinoma-associated increases in EPRS, which was also positively associated with LRPPRC and COPG1 (Fig. 1). EPRS was repeatedly elevated in adenocarcinoma relative to control in 79% of cancer subjects, whereas both LRPPRC and COPG1 were consistently elevated in 76% of subjects (Table 2). Adenocarcinoma-dependent elevations in LRPPRC were associated with similar increases in STOML2 and PDIA4, which exhibited 2- and 2.3-fold increases in adenocarcinoma relative to control tissue, respectively (Fig. 1; Table 2). NANS, COPG1 and PDIA4 were all negatively associated with adenocarcinoma-dependent reductions in PTRF, which was generally decreased in adenocarcinoma compared to control tissue (89% of patients).

mRNA expression data from the Okayama et al. study on NSCLC adenocarcinoma [17] was used to strengthen the adenocarcinoma-associated proteomic perturbations. Only Stage I NSCLC adenocarcinomas were considered. Consistent with our proteomic data, mRNA expression of *APEX1*, *HYOU1*, *PDIA4*, *NANS*, *LRPPRC*, *EPRS* and *COPG1* were significantly (Mann–Whitney U < 0.05) higher in adenocarcinoma compared to control whereas mRNA abundance of *HBG1*, *HBG2*, *HBD*, and *PTRF* were significantly (Mann–Whitney U < 0.05) lower (Additional file 1: Figure S1). No significant differences were observed for *SCL4A1*, *SPTB, SPTA1* and *ANK1*. *STOML2* was not detected in the Okayama Lung dataset.

### Validation of HYOU1 and EPRS by immunohistochemistry of LC003 NSCLC adenocarcinoma TMA

Immunohistochemistry of a tissue microarrays (LC003 TMA) for NSCLC adenocarcinoma and matched controls (n = 40) was used to validate our proteomic findings. We focused on EPRS and HYOU1 due to both proteins being in the top 10% features that distinguish malignant from control tissue and due to their positive staining based on the Cancer Protein Atlas. All TMA results were blindly scored using s manual “H” scoring system (“Methods”).

**Fig. 2:**
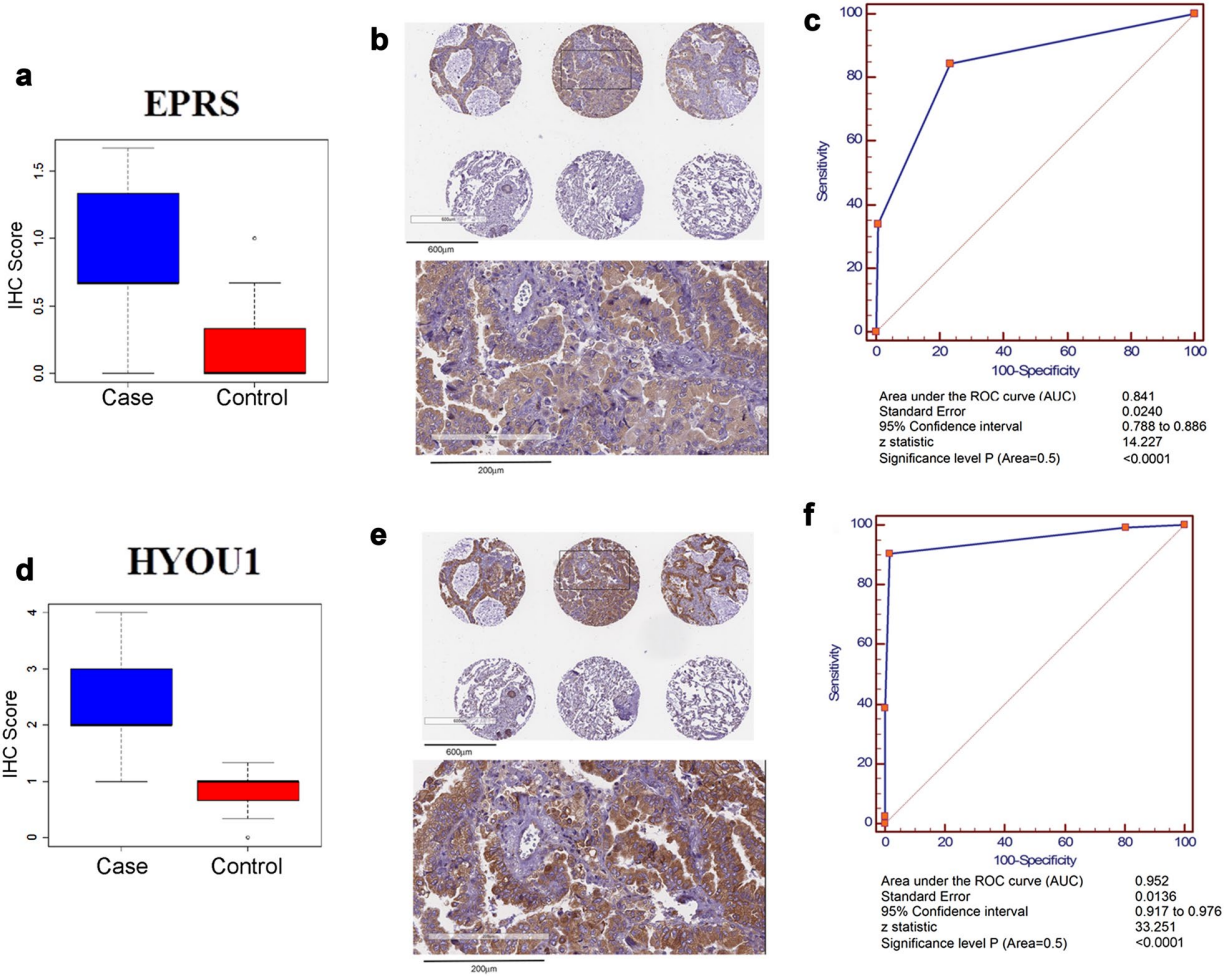
TMA Validation of EPRS and HYOU1. **a** IHC scores for EPRS in TMA. **b** Representative IHC sections of EPRS in adenocarcinoma and control. **c** EPRS receiver operating characteristic (ROC) curve and statistical analysis for all 40 cases and controls using results from the LC003 TMA. **d** IHC scores for HYOU1 in TMA. **e** Representative IHC sections of HYOU1 in adenocarcinoma and control. **f** Receiver operating characteristic (ROC) curve for HYOU1 in TMA

EPRS and HYOU1 immunostaining were elevated in adenocarcinoma relative to control tissue (Fig. 2). Immunostaining revealed EPRS cytoplasmic expression in 36 (90%) cases. Of 36 cases, medium expression was observed in 11 (30.5%) cases (Additional file 1: Figure S2). Twenty-five (69.5%) adenocarcinoma cases showed low expression (Additional file 1: Figure S2). Representative sections of control tissue and adenocarcinoma are provided in Fig. 2b. EPRS exhibited an AUC of 0.841 (CI 0.788–0.866; p < 0.001) for classifying tissue as malignant. HYOU1 was expressed in all 40 (100%) cases of adenocarcinoma (Fig. 2d). Representative sections of control tissue and adenocarcinoma are provided in Fig. 2e. Macrophages and rare reactive pneumocytes in control tissue demonstrated weak staining (Fig. 2e). The staining of HYOU1 in adenocarcinoma cells was localized in the cytoplasm (Fig. 2e). HYOU1 staining was high in 14 cases (35%), medium in 21 cases (52.5%) and low in 5 cases (12.5%) (Additional file 1: Figure S2) and exhibited an AUC of 0.952 (CI 0.917–0.976; p < 0.001) for classifying tissue as malignant.

### Association between proteomic signatures and overall survival

**Fig. 3:**
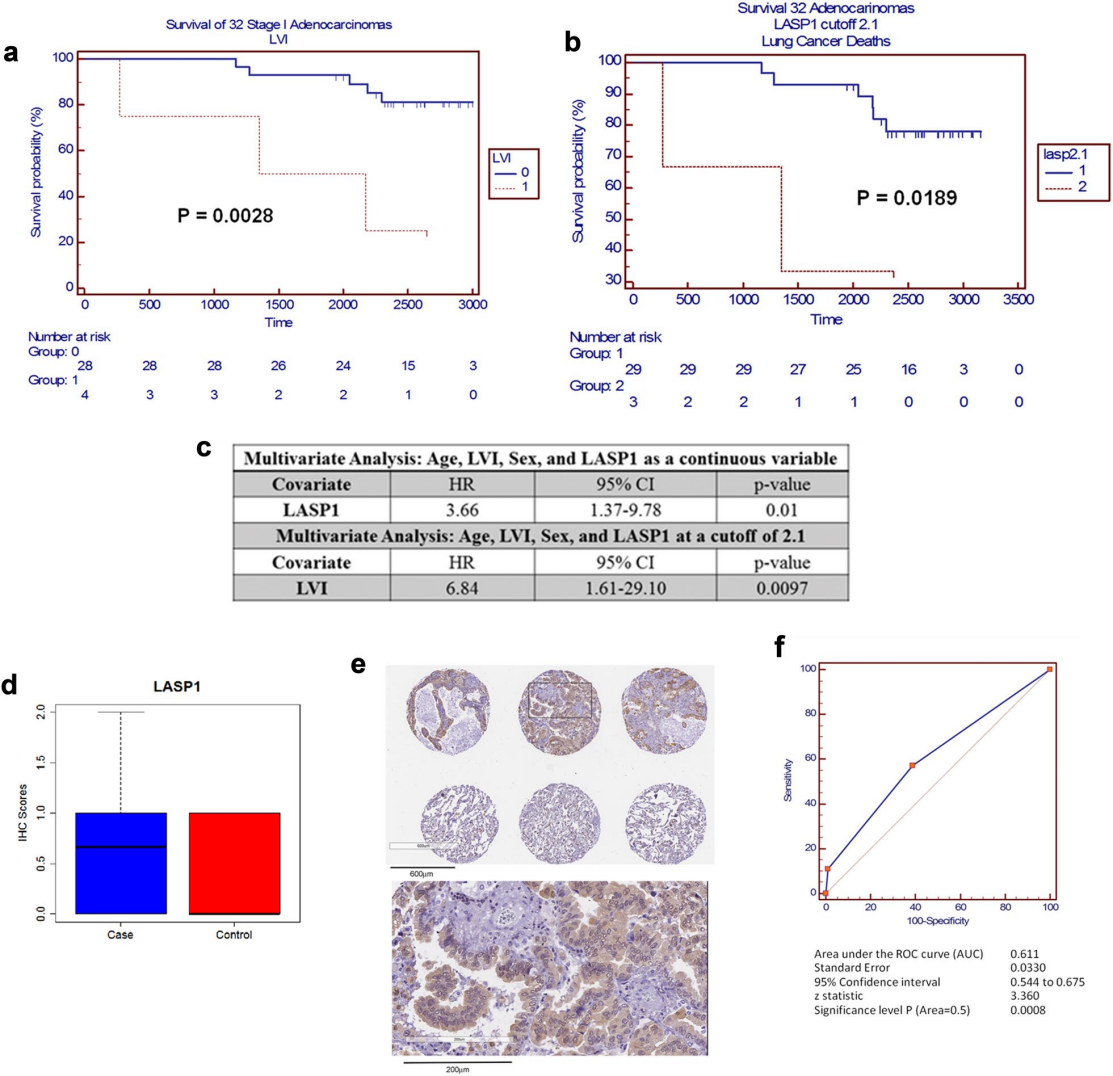
Association between LASP1, lymphovascular infiltration and survival. Kaplan–Meier survival curves are shown for subjects stratified presence of lymphovascular infiltration (LVI) (**a**) or by LASP1 protein abundance cutoff of 2.1 (**b**). **c** Multivariate Cox proportional hazard ratios are shown for LASP1 as a continuous variable and LASP1 with a cutoff of 2.1 spectral counts. Only LASP1 was a significant independent risk factor for overall survival when evaluated as a continuous variable but not at optimal x-tile derived cutoff of 2.1 spectral counts when accounting for other co-variants. **d** IHC scores for LASP1 in TMA. **e** Representative IHC sections of LASP1 in adenocarcinoma and control. **f** Receiver operating characteristic (ROC) Curve for LASP1 in TMA

Lymphovascular invasion (LVI) is a negative prognostic factor for development of distant metastasis and long-term survival in NSCLC, particularly in early stage lung adenocarcinoma [19, 20]. LVI was significantly associated with poor overall survival in our cohort (Fig. 3a). We further probed whether our proteomic findings would provide prognostic value independent of LVI as part of the discovery phase. All proteins were considered for this analysis. Six individuals of the 38 subjects from our cohort were excluded from the analysis as their deaths were not due to cancer. We identified LASP1 as a negative predictor of overall survival (Fig. 3b). Multivariate Cox proportional hazard models were used to determine hazard ratios between LASP1 + LVI + Age + Gender and overall survival. LASP1 as a continuous variable was determined to be an independent prognostic factor for overall survival (hazard ratio of 3.66 [CI 1.37–9.78; p = 0.01)] when including LVI + Age + Gender as covariables (Fig. 3c). However, only LVI was a significant prognostic factor for overall survival when an x-tile [21] derived optimum LASP1 cutoff value of 2.1 spectral counts was used (HR 6.84; CI 1.61–29.10; p = 0.0097) (Fig. 3c). When considering subjects that died as a consequence of NSCLC adenocarcinoma based on our proteomic findings an AUC of 0.755 (CI 0.572–0.889; p = 0.0107) was determined (Additional file 1: Figure S3). Additionally, TMA results indicated that most adenocarcinoma cases revealed low expression of LASP1 immunostaining (18/23, 78.2%) (Fig. 3d; Additional file 1: Figure S3). Representative sections of control tissue and adenocarcinoma from the TMA are provided in Fig. 3e. Overall LASP1 indicated moderate classification performance with an AUC of 0.611 (CI = 0.544–0.675) (Fig. 3f). Survival information was not available for TMA results.

To further evaluate the association between LASP1 and overall survival we utilized *LASP1* mRNA data from Okayama et al. [17] study on NSCLC adenocarcinoma. Only stage I NSCLC adenocarcinomas were considered. Multivariate Cox proportional hazard models including Gender + Age + Smoking Status + *LASP1* mRNA as a continuous variable indicated that *LASP1* mRNA abundance was a significant negative predictor of overall survival per unit increase (HR 9.948; CI 8.931–10.965; p < 0.001) (Table 3). Kaplan–Meier survival curves for *LASP1* stratified by quantiles are shown in Additional file 1: Figure S4. These findings coincide with our proteomic findings and highlight the potential of LASP1 as a candidate prognostic marker for early stage NSCLC adenocarcinoma.

**Table 3:** Hazard ratios for *LASP1* mRNA abundance and overall survival in Okayama Dataset

Variable	Univariate			Multivariate		
	Hazard ratio	95% CI	p value	Hazard ratio	95% CI	p value
Gender						
Female	Reference			Reference		
Male	1.333	(0.38–2.286)	0.55	0.953	(–0.35–2.256)	0.94
Age						
≤61 years of age	Reference			Reference		
>61 years of age	1.674	(0.708–2.64)	0.3	1.865	(0.889–2.841)	0.21
Smoking						
Never smoker	Reference			Reference		
Smoker	1.699	(0.746–2.652)	0.28	1.509	(0.21–2.808)	0.53
*LASP1* ^a^	9.567	(8.571–10.563)	<0.001	9.948	(8.931–10.965)	<0.001

^a^Per unit increase

## Discussion

In the current study, we evaluated the proteome of 38 malignant and matched control tissue of stage IA and IB lung adenocarcinoma. Differential analysis identified 436 differentially expressed proteins in adenocarcinoma compared to control tissue, of which 367 remained significant following false-discovery rate adjustment. Orthogonal partial least squares discriminant analysis identified the top 10% proteins that significantly differed between adenocarcinoma and control tissue. A Gaussian graphical model network was used to identify conditionally independent empirical protein–protein relationships between O-PLS-DA selected discriminants for adenocarcinoma. Of the 16 distinguishing proteins, 8 were significantly elevated in lung adenocarcinoma (COPG1, STOML2, HYOU1, PDIA4, EPRS, APEX1, LRPPRC and NANS) whereas 8 proteins were significantly decreased in lung adenocarcinoma (SPTB, SPTA1, ANK1, SLC4A1, HBG1 and HBG2). A subsequent sub-analysis was conducted to further evaluate the prognostic capacity of using all identified proteins.

### Top discriminatory proteins of adenocarcinoma indicate heightened intrinsic defense mechanisms, altered metabolism and perturbed membrane integrity

NANS (N-acetylneuraminic acid synthase) exhibited the largest increased (2.7-fold) in lung adenocarcinoma relative to control and was consistently elevated in 82% of the subjects. The elevation in NANs suggests increased biosynthesis of sialic acid, which is known to be elevated in many cancers and may exhibit immune-modulatory and anti-apoptotic functions [22]. Elevation in NANS was positively correlated with adenocarcinoma-associated elevations in APEX1 (apurinicapyrimidinic endonuclease), an important component of base excision repair (BER) pathway and transcriptional modulator of genes that protect against oxidative stress [23]. Over expression of APEX1 has been described in NSCLC and other cancers [23], while its down-regulation may induce apoptosis [24]. The elevation in APEX1 is particularly interesting since radiotherapy/chemotherapy induce DNA damage and promote production of cytotoxic reactive oxygen species (ROS) [25]. An increase in repair machinery and anti-oxidant defense systems within a tumor cell could interfere with these treatments. APEX1 has been linked to chemotherapy/radiotherapy resistance [26].

The elevation in APEX1 was paralleled by an increase in HYOU1, a heat shock protein, which has important roles in hypoxia and angiogenesis and is linked to tumor prognosis [27]. Increased HYOU1 also positively correlated with PDIA4, a known modulator of redox status, which has been shown to promote drug resistance to cisplatin in lung adenocarcinoma [28, 29]. PDIA4 also directly correlated to LRPPRC. LRPPRC acts as a regulator of mitochondrial DNA-encoded mRNAs and participates in glucose homeostasis, energy metabolism and nuclear receptor activation [30, 31] and is abundantly expressed in NSCLC adenocarcinoma and other cancers [30]. Knockdown of LRPPRC promotes apoptosis and reduces tumor invasiveness [30]. Elevation in LRPPR was paralleled by increases in STOML2 and COPG1. STOML2, a member of the stomatin family, is upregulated in numerous cancers and linked to tumor aggressiveness [32]. COPG1 is a component of the COPI complex, an integral component in lipid homeostasis that promotes lipolysis through the association of PNPLA2, a triglyceride lipase, with lipid droplets [33]. Lipid metabolism is known to be perturbed in various malignancies acting as energy sources, signaling moieties and for biosynthesis of structural membrane lipids facilitating increased proliferative potential [34]. Cancer cells not only rely on *de novo* fatty acid synthesis, but also take fatty acids from their surrounding microenvironment [34]. We previously reported that malignant cells exhibit perturbations in free fatty acid profiles with most fatty acids being decreased in tumorous tissue, with the exception of arachidonic acid that was elevated in tumor compared to non-tumor tissue [16]. Free fatty acids, particularly arachidonic acid, serve as substrates for generation of inflammatory mediators. This may also provide a basis for the tumor-associated elevation in EPRS, which was positively correlated with LRPPRC and COPG1. EPRS (glutamyl-prolyl-tRNA synthetase) is a bifunctional enzyme thought to be a gatekeeper of inflammatory gene translation, modulator of angiogenesis and regulator of amino-acid stress responses [35]. EPRS is also regulated by c-Myc, which is commonly amplified in lung adenocarcinoma and modulates tumor metabolism [36, 37]. Collectively, increases in LRPRRC, COPG1, EPRS and STOML2 all point towards alterations in energetics, metabolism and alterations in inflammatory responses that accompany transformation.

Adenocarcinoma-associated reductions in spectrins, SPTB and SPTA1, and ankrin (ANK1) all interact with each another to regulate cell shape and membrane integrity, so paralleled changes likely reflect changes in cell adherence, which is a known hallmark of metastatic cancer [38]. Alterations in SPTB and ANK1 also indicate changes in rearrangement of transmembrane proteins, including ion channels, which may account for the observed positive correlation between SPTB and SLC4A1, a membrane bound anion exchange transporter. SLC4A1 plays central roles in pH homeostasis and has been linked to tumor aggressiveness in numerous cancer types [39]. Since most cancer cells exhibit a metabolic shift towards acidic-producing pathways, reflective of both oncogenic signaling and hypoxia, upregulation of pH-regulatory transport proteins may be important [39]. An alteration in SLC4A1 represents an attractive target for therapeutic intervention and may provide diagnostic value.

### LASP1 as a potential negative prognostic indicator for overall survival

LIM and SH3 domain protein (LASP1) a dynamic protein involved in cell structure, physiological processes and cell signaling, is significantly expressed in various malignancies and associated with tumor aggressiveness [40] and is reported to be an independent prognostic factor in patient’s survival for gastric cancer [41] and hepatocellular carcinoma [42]. We also found that LASP1 was associated with poor overall survival in our cohort. When considered as a continuous variable, LASP1 indicated a HR of 3.66 (CI 1.37–9.78; p 0.01) per unit increase for increased risk of death. Notably, adenocarcinoma abundance of LASP1 was only significantly higher relative to control tissue in those subjects who died (Additional file 1: Table S4). To strengthen the association between LASP1 and overall survival, we utilized a second independent dataset on *LASP1* mRNA expression in NSCLC adenocarcinoma [17]. *LASP1* mRNA expression was a significant negative predictor of overall survival. LASP1 has been shown to promote invasion and metastasis; particularly, LASP1 overexpression in pancreatic ductal adenocarcinomas was found to be significantly associated with lymph node metastasis and poor overall survival [43]. Therefore, it is plausible that tumor-associated elevations in LASP1 may contribute to LVI, which could explain why we no longer observed a significant hazard ratio when a LASP1 cutoff of 2.1 was used and instead only LVI was found to be a significant predictor of overall survival. Collectively, these findings highlight the potential of LASP1 as a prognostic indicator for NSCLC adenocarcinoma. Further studies in larger cohorts are required to fully validate these findings.

One limitation of the current study, and others like it, was the lack of assessment of tissue microheterogeneity at the sub-biopsy level. We acknowledge that changes in specific proteins, such as hemoglobin subunits, may reflect contamination from red blood cells. However, changes in erythrocyte-associated proteins may equally reflect altered tumor angiogenesis [44]. Chen et al. used 2D-PAGE, MALDI MS to analyze Stage I and III lung adenocarcinomas and found proteins that were heavily implicated in antioxidant response systems and cellular metabolism [45] consistent with our findings. Zhou et al. used 2D-DIGE with followed-up by TMA IHC and blood studies of TyrRS and MACF-1 [46]. However, Zhou et al. evaluated tissues from stage II and III lung adenocarcinoma and thus might not be directly compared to our results for stage IA or IB given the inherent heterogeneity that exists among different tumor stages. Kikuchi et al. conducted in-depth proteomic profiling on lung adenocarcinomas and squamous cell carcinomas and compared these against control tissue [47]. Similar to our findings, Kikuchi et al. also found tumor-associated elevations in numerous mentioned above proteins including HYOU1, NANS and PDIA4 [47].

In conclusion, proteomic changes in early stage NSCLC adenocarcinoma tissues are consistent with known cancer-dependent alterations in repair machinery, redox status, energetics, and inflammation. The current study identified candidate markers that may help identify at-risk subjects and assist with treatment. This study also suggests that LASP1 might serve as a potential negative prognostic marker for overall survival. Further studies in larger cohorts are warranted to confirm and validate these findings.

## Additional file

**Additional file 1.** Supplemental Materials and Methods 1a–1d. **Table S2.** Summary of changes for significantly (pFDR < 0.05) differential proteins between cancer and control tissue. **Table S3**. OPLSDA model validation statistics. **Table S4**. Associations between LASP1 and lymphovascular infiltration and overall survival. **Table S5**. Patient characteristics for oncomine Okayama lung study. **Figure S1**. mRNA expression of top candidates in independent dataset of early stage adenocarcinoma relative to control. **Figure S2**. Immunohistochemistry scores for EPRS, HYOU1 and LASP1 in LC003 TMA. **Figure S3**. Receiver operating characteristic curve for LASP1 in subjects who died from NSCLC adenocarcinoma. **Figure S4**. Kaplan-Meier survival curves for LASP1 mRNA expression quantiles in oncomine Okayama lung cancer study.

**Additional file 2. Table S1.** Identified proteins.
