# Supplementary material for: Proteomic profiling of lung adenocarcinoma indicates heightened DNA repair, antioxidant mechanisms and identifies LASP1 as a potential negative predictor of survival
Source: Clin Proteomics. 2016 Oct 27;13:31. doi: 10.1186/s12014-016-9132-y (PMC5084393; doi:10.1186/s12014-016-9132-y)
Supplement: Supplementary file 1 — Additional file 1. Supplemental Materials and Methods 1a–1d. Table S2. Summary of changes for significantly (pFDR < 0.05) differential proteins between cancer and control tissue. Table S3. OPLSDA model validation statistics. Table S4. Associations between LASP1 and lymphovascular infiltration and overall survival. Table S5. Patient characteristics for oncomine Okayama lung study. Figure S1. mRNA expression of top candidates in independent dataset of early stage adenocarcinoma relative to control. Figure S2. Immunohistochemistry scores for EPRS, HYOU1 and LASP1 in LC003 TMA. Figure S3. Receiver operating characteristic curve for LASP1 in subjects who died from NSCLC adenocarcinoma. Figure S4. Kaplan-Meier survival curves for LASP1 mRNA expression quantiles in oncomine Okayama lung cancer study. [file 12014_2016_9132_MOESM1_ESM.docx]

**Proteomic Profiling of Lung Adenocarcinoma Indicates Heightened DNA Repair, Antioxidant Mechanisms and Identifies LASP1 as a Potential Negative Predictor of Survival**

Johannes F. Fahrmann^1^*, Dmitry Grapov^2^*, Brett S. Phinney^3^, Carol Stroble^4^, Brian C. DeFelice^1^, William Rom^5^, David R. Gandara^4^, Yanhong Zang^6^, Oliver Fiehn^1,7^, Harvey Pass^8^, Suzanne Miyamoto^4#^

***EQUAL CONTRIBUTORS**

^1^ University of California, Davis Genome Center, Davis CA

^2^ CDS Creative Data Solutions, Ballwin, MO

^3^ Genome Center Proteomics Core Facility, Davis CA

^4^ Division of Hematology and Oncology, Department of Internal Medicine, School of Medicine, University of California, Davis Medical Center, Sacramento, CA 91817

^5^ Division of Thoracic Surgery, Department of Cardiothoracic Surgery, Langone Medical Center, New York University, New York City, NY, USA

^6^Department of Pathology and Laboratory Medicine, University of California, Davis Medical Center, Sacramento CA 95817

^7^ Department of Biochemistry, Faculty of Sciences, King Abdulaziz University, Jeddah 21589, Saudi-Arabia

^8^Division of Pulmonary, Critical Care, and Sleep, NYU School of Medicine, New York, NY, USA

**Running Title:** Proteomic Perturbations in Early Stage Lung Adenocarcinoma

#Corresponding Author:

Suzanne Miyamoto

Division of Hematology and Oncology, Department of Internal Medicine

4501 X Street, Suite 3016, Sacramento, CA 95817

Phone: 916-734-3769

Fax: 916-734-5356

[smiyamoto@ucdavis.edu](mailto:smiyamoto@ucdavis.edu)

**Supplemental Materials and Methods**

**1a. LC-MS/MS Analysis**

Samples were block randomized into 8 blocks of 4 normal and 4 tumor samples in each block. Three technical replicates were acquired for each sample resulting in 192 total LC-MS/MS analyses. LC separation was done on a Waters Nano Acquity UHPLC (Waters Corporation) with a Proxeon nanospray source.

The digested peptides were reconstituted in 2% acetonitrile/0.1% trifluoroacetic acid and roughly 3µg of each sample was loaded onto a 100 micron x 25 mm Magic C18 100Å 5U reverse phase trap where they were desalted online before being separated on a 75 micron x 150 mm Magic C18 200Å 3U reverse phase column. Peptides were eluted using a gradient of 0.1% formic acid (A) and 100% acetonitrile (B) with a flow rate of 300nL/min. A 120 minute gradient was ran with 5% to 35% B over 100 minutes, 35% to 80% B over 8 minutes, 80% B for 1 minute, 80% to 5% B over 1 minute, and finally held at 5% B for 10 minutes. Each of the gradients was followed by a 1h column wash.

Mass spectra was collected on an Orbitrap Q Exactive Plus mass spectrometer (Thermo Fisher Scientific) in a data-dependent mode with one MS precursor scan followed by 15 MS/MS scans. A dynamic exclusion of 15 seconds was used. MS spectra were acquired with a resolution of 70,000 and a target of 1 × 10^6^ ions or a maximum injection time of 30ms. MS/MS spectra were acquired with a resolution of 17,500 and a target of 5 × 10^4^ ions or a maximum injection time of 50ms. Peptide fragmentation was performed using higher-energy collision dissociation (HCD) with a normalized collision energy (NCE) value of 27. Unassigned charge states as well as +1 and ions >+5 were excluded from MS/MS fragmentation.

**1b. Protein Identification**

Raw mass spectrometry files were converted to mzML using MSConvert and peptide spectrum matching (PSM) was performed using MS-GF+ (version 1.0072) and Myrimatch (version 2.2.140) against the NCBI Refseq human database (72,090 sequences, October 2014) plus 110 additional sequences of common laboratory contaminants from the cRAP protein database ([www.thegpm.org/crap](http://www.thegpm.org/crap)) plus an equal number of reverse decoy sequences. Enzyme specificity was set to Trypsin/P with dynamic modifications of deamidation of NQ and oxidation of M. A static modification was set to Carbamidomethyl C. Precursor mass tolerance was set to 10 ppm for Myrimatch and 20 ppm for MS-GF+, fragment ion tolerance was set to 20 ppm for both search engines. Myrimatch and MS-GF+ identifications were combined and PSM’s were assembled into proteins using IDPicker 3. Proteins were assembled on the gene level and IdPicker3 parameters were set to the following. Max Q value = 0.005, minimum peptides per gene = 2, minimum spectra per gene = 4. Minimum spectra per match =1, Max Protein Groups per Peptide =10. These parameters resulted in 79,775 distinct peptide matches corresponding to 4,512,957 spectra assembled into 6,796 gene groups and 10,712 protein groups. A decoy false discovery rate was calculated on the protein, peptide and spectrum level. Protein FDR= 6.6%, Peptide FDR = 5%, Spectrum FDR = 0.29%.

**1c. Immunohistochemistry of TMA sections**

Briefly, slides were deparaffinized and pretreated with Heat-Induced-Epitope-Retrieval solution (pH 9) (DAKO). Slides were preincubated in Dual Endogenous Enzyme Block (DEEB) blocking solution in a DAKO Autostainer Link48. Polyclonal anti-human HYOU1 (1:100, HPA049296, SIGMA), EPRS (1:100, HPA030052, SIGMA) and LASP1 (1:500, HPA012072, SIGMA) were incubated for 30 minutes and then washed, which was followed by detection by EDL solution (DAKO EnVision + Dual Link System-HRP). Hematoxylin was used as the counterstain.

**1d. Statistical Analysis**

Replicate precision as relative standard deviation (RSD) was compared between protein spectral counts and area under the curve measurements. Protein count data displayed lower median RSD for the majority of measured proteins and was selected for all subsequent analyses. Count data for 10712 proteins was evaluated for missing or zero values, replicate precision, protein sequence coverage and annotation to know genes. Robust measurements were retained based on median counts > 0 for control or malignant tissue and protein sequence coverage > 60% were used to limit interpretation to only the most abundant, robust and reproducible proteomic measurements. Analysis of replicate precision was used to filter multiple measurements mapping to the same gene to select variables with lowest RSD. Data normalization strategies were compared based on RSD. Mean normalized count data (each sample count expressed as a ratio to the total counts per sample) yielded lowest RSD and was selected for further analyses. Based on the outlined strategy, 799 high quality protein measurements or 14% of the initially measured features were selected for further statistical and multivariate analyses.

Orthogonal partial least squares discriminant analysis (O-PLS-DA) multivariate models were used to identify the top 10% of all protein discriminants between tumor and control tissues. O-PLS-DA modeling was conducted on shifted natural logarithm transformed age, gender and packs adjusted mean normalized protein counts. Double cross-validation was used for model optimization and validation. One third of the sample set (based on patients) was held out for a test set. The remaining two thirds of the data were used for feature selection based model optimization. Filter based feature selection on full model variable loadings was used to identify the optimal proteomic feature set based on Monte Carlo cross-validated (MCCV) (100 replications) model area under the receiver operator characteristic curve (AUC), sensitivity (Sens) and specificity (Spec) (19).

A Gaussian graphical model protein-protein empirical network was calculated for O-PLS-DA selected top discriminants (n= 16) as previously described [16]. Partial correlations were calculated using q-order partial correlation analysis of shifted natural logarithm transformed covariate adjusted and mean normalized protein counts [16]. Conditionally independent protein-protein associations were identified based on false-discovery rate (FDR) adjusted p-value <0.05.

**Supplemental Tables**

**Table S2.** Summary of changes for significantly (pFDR <0.05) differential proteins between cancer and control tissue.

**Table S3.** OPLSDA Model Validation Statistics.

**Table S4.** Associations between LASP1 and lymphovascular infiltration and overall survival.

**Table S5.** Patient Characteristics for Oncomine Okayama Lung Study.

**Table S2.** Summary of changes for significantly (pFDR <0.05) differential proteins between cancer and control tissue.

| Gene ID | Gene Name | Tumor^a^ | Normal^a^ | Fold Change^b^ | p-value^c^ | Ratio^d^ | Percent (%)^e^ |
| --- | --- | --- | --- | --- | --- | --- | --- |
| SPTB | spectrin, beta, erythrocytic | 2.82 +/- 2.6 | 10.7 +/- 9.3 | 0.3 | 6.7E-07 | (7/38) | 18 |
| AGR2 | anterior gradient 2 | 4.33 +/- 3.1 | 1.41 +/- 0.8 | 3.1 | 6.7E-07 | (34/38) | 89 |
| EHD1 | EH-domain containing 1 | 2.55 +/- 1.9 | 5.13 +/- 2.7 | 0.5 | 0.0000025 | (5/38) | 13 |
| EPRS | glutamyl-prolyl-tRNA synthetase | 3.15 +/- 1.7 | 1.24 +/- 0.87 | 2.5 | 0.0000025 | (30/38) | 79 |
| GDI2 | GDP dissociation inhibitor 2 | 6.02 +/- 2.5 | 3.24 +/- 2 | 1.9 | 0.0000031 | (30/38) | 79 |
| SPTA1 | spectrin, alpha, erythrocytic 1 | 4.28 +/- 3.4 | 13.3 +/- 12 | 0.3 | 0.0000031 | (8/38) | 21 |
| ACO2 | aconitase 2, mitochondrial | 4.78 +/- 1.9 | 2.41 +/- 1.6 | 2 | 0.000004 | (31/38) | 82 |
| IDH1 | isocitrate dehydrogenase 1 (NADP+), soluble | 3.52 +/- 1.6 | 1.54 +/- 1.3 | 2.3 | 0.000004 | (31/38) | 82 |
| ACO1 | aconitase 1, soluble | 3.39 +/- 2.1 | 1.45 +/- 1.2 | 2.3 | 0.000004 | (31/38) | 82 |
| PTBP1 | polypyrimidine tract binding protein 1 | 6 +/- 2.1 | 3.32 +/- 1.8 | 1.8 | 0.0000044 | (31/38) | 82 |
| GANAB | glucosidase, alpha; neutral AB | 8.03 +/- 3 | 4.2 +/- 2.4 | 1.9 | 0.0000046 | (33/38) | 87 |
| HSPA9 | heat shock 70kDa protein 9 (mortalin) | 5.04 +/- 2.5 | 2.62 +/- 1.8 | 1.9 | 0.0000046 | (30/38) | 79 |
| XRCC5 | X-ray repair complementing defective repair in Chinese hamster cells 5 (double-strand-break rejoining) | 5.28 +/- 1.9 | 2.82 +/- 1.8 | 1.9 | 0.0000055 | (32/38) | 84 |
| ENO2 | enolase 2 (gamma, neuronal) | 5.7 +/- 3.4 | 2.12 +/- 1.4 | 2.7 | 0.0000055 | (31/38) | 82 |
| IDH2 | isocitrate dehydrogenase 2 (NADP+), mitochondrial | 3.58 +/- 1.4 | 1.66 +/- 1.3 | 2.2 | 0.0000082 | (33/38) | 87 |
| ATIC | 5-aminoimidazole-4-carboxamide ribonucleotide formyltransferase/IMP cyclohydrolase | 3.18 +/- 1.8 | 1.41 +/- 0.98 | 2.3 | 0.000012 | (29/38) | 76 |
| EEF1A1 | eukaryotic translation elongation factor 1 alpha 1 | 8.55 +/- 3.2 | 4.63 +/- 2.6 | 1.8 | 0.000012 | (31/38) | 82 |
| ENO1 | enolase 1, (alpha) | 24 +/- 13 | 9.72 +/- 6.6 | 2.5 | 0.000013 | (30/38) | 79 |
| NAMPT | nicotinamide phosphoribosyltransferase | 3.53 +/- 2.2 | 1.44 +/- 1.1 | 2.4 | 0.000014 | (28/38) | 74 |
| SND1 | staphylococcal nuclease and tudor domain containing 1 | 5.13 +/- 2.1 | 2.48 +/- 2.1 | 2.1 | 0.000015 | (32/38) | 84 |
| SLC4A1 | solute carrier family 4 (anion exchanger), member 1 (Diego blood group) | 3.8 +/- 2.7 | 8.75 +/- 5.2 | 0.4 | 0.000015 | (9/38) | 24 |
| PYGB | phosphorylase, glycogen; brain | 4.58 +/- 2.6 | 2.26 +/- 1.1 | 2 | 0.000028 | (31/38) | 82 |
| LGALS3BP | lectin, galactoside-binding, soluble, 3 binding protein | 3.36 +/- 1.9 | 1.6 +/- 1.2 | 2.1 | 0.000029 | (28/38) | 74 |
| SDPR | serum deprivation response | 1.74 +/- 1.5 | 3.56 +/- 1.6 | 0.5 | 0.000029 | (5/38) | 13 |
| CNDP2 | CNDP dipeptidase 2 (metallopeptidase M20 family) | 6.2 +/- 2.2 | 3.71 +/- 2 | 1.7 | 0.000033 | (32/38) | 84 |
| PKM | pyruvate kinase, muscle | 13.9 +/- 7 | 7.04 +/- 3.9 | 2 | 0.000034 | (30/38) | 79 |
| HBG2 | hemoglobin, gamma G | 7.43 +/- 6.2 | 17 +/- 9.7 | 0.4 | 0.000035 | (9/38) | 24 |
| SFN | stratifin | 2.73 +/- 1.6 | 1.19 +/- 0.88 | 2.3 | 0.000036 | (27/38) | 71 |
| HBG1 | hemoglobin, gamma A | 7.44 +/- 6.2 | 16.9 +/- 9.7 | 0.4 | 0.000038 | (9/38) | 24 |
| GARS | glycyl-tRNA synthetase | 1.68 +/- 1.2 | 0.544 +/- 0.51 | 3.1 | 0.000039 | (32/38) | 84 |
| STOM | stomatin | 2.66 +/- 2 | 4.79 +/- 2.1 | 0.6 | 0.00004 | (6/38) | 16 |
| EEF2 | eukaryotic translation elongation factor 2 | 13.1 +/- 5.9 | 7.04 +/- 3.9 | 1.9 | 0.00004 | (31/38) | 82 |
| HYOU1 | hypoxia up-regulated 1 | 4.74 +/- 2.3 | 2.12 +/- 1.9 | 2.2 | 0.00004 | (31/38) | 82 |
| TKT | transketolase | 10.7 +/- 5.5 | 5.03 +/- 3.6 | 2.1 | 0.00004 | (28/38) | 74 |
| GFPT1 | glutamine--fructose-6-phosphate transaminase 1 | 1.89 +/- 1.6 | 0.669 +/- 0.35 | 2.8 | 0.00004 | (30/38) | 79 |
| AK2 | adenylate kinase 2 | 2.5 +/- 1.2 | 1.06 +/- 1.1 | 2.4 | 0.00004 | (32/38) | 84 |
| APEX1 | APEX nuclease (multifunctional DNA repair enzyme) 1 | 2.05 +/- 1.1 | 0.778 +/- 0.83 | 2.6 | 0.000044 | (32/38) | 84 |
| PECAM1 | platelet/endothelial cell adhesion molecule 1 | 1.55 +/- 1.4 | 3.19 +/- 1.6 | 0.5 | 0.000056 | (6/38) | 16 |
| ERP29 | endoplasmic reticulum protein 29 | 2.23 +/- 1.6 | 0.901 +/- 0.82 | 2.5 | 0.000056 | (29/38) | 76 |
| SPTBN1 | spectrin, beta, non-erythrocytic 1 | 12.5 +/- 8.7 | 23.7 +/- 7.9 | 0.5 | 0.000059 | (6/38) | 16 |
| ANK1 | ankyrin 1, erythrocytic | 1.84 +/- 1.8 | 6.28 +/- 5.7 | 0.3 | 0.000067 | (8/38) | 21 |
| AKR1A1 | aldo-keto reductase family 1, member A1 (aldehyde reductase) | 2.71 +/- 1.5 | 1.24 +/- 1.1 | 2.2 | 0.000067 | (29/38) | 76 |
| S100A10 | S100 calcium binding protein A10 | 3.01 +/- 2.1 | 5.15 +/- 2.3 | 0.6 | 0.000068 | (7/38) | 18 |
| SOD2 | superoxide dismutase 2, mitochondrial | 3.02 +/- 1.7 | 1.45 +/- 1 | 2.1 | 0.00007 | (28/38) | 74 |
| ARHGDIB | Rho GDP dissociation inhibitor (GDI) beta | 3.7 +/- 1.9 | 1.94 +/- 1.2 | 1.9 | 0.00007 | (27/38) | 71 |
| AARS | alanyl-tRNA synthetase | 1.87 +/- 1.4 | 0.695 +/- 0.5 | 2.7 | 0.00007 | (30/38) | 79 |
| PTRF | polymerase I and transcript release factor | 3.81 +/- 2.8 | 7.35 +/- 2.8 | 0.5 | 0.00007 | (4/38) | 11 |
| PPIA | peptidylprolyl isomerase A (cyclophilin A) | 6.68 +/- 2.8 | 4.21 +/- 2.5 | 1.6 | 0.000074 | (26/38) | 68 |
| PARK7 | parkinson protein 7 | 5.28 +/- 2.3 | 2.69 +/- 2.1 | 2 | 0.000076 | (28/38) | 74 |
| PGAM1 | phosphoglycerate mutase 1 (brain) | 3.51 +/- 2.2 | 1.81 +/- 1.4 | 1.9 | 0.00008 | (29/38) | 76 |
| TNS1 | tensin 1 | 3.44 +/- 2.1 | 5.66 +/- 2.5 | 0.6 | 0.000083 | (6/38) | 16 |
| PGM1 | phosphoglucomutase 1 | 2.5 +/- 1.5 | 1.11 +/- 1.1 | 2.2 | 0.000083 | (29/38) | 76 |
| TYMP | thymidine phosphorylase | 4.02 +/- 2.2 | 2.19 +/- 1.4 | 1.8 | 0.000083 | (28/38) | 74 |
| UGDH | UDP-glucose 6-dehydrogenase | 2.75 +/- 1.8 | 1.29 +/- 1.2 | 2.1 | 0.000091 | (32/38) | 84 |
| SPTAN1 | spectrin, alpha, non-erythrocytic 1 | 19.2 +/- 12 | 33.4 +/- 10 | 0.6 | 0.0001 | (7/38) | 18 |
| HBD | hemoglobin, delta | 21.7 +/- 18 | 47.3 +/- 32 | 0.5 | 0.00013 | (8/38) | 21 |
| LRPPRC | leucine-rich pentatricopeptide repeat containing | 3.52 +/- 2.5 | 1.31 +/- 1.4 | 2.7 | 0.00013 | (29/38) | 76 |
| TUFM | Tu translation elongation factor, mitochondrial | 3.62 +/- 1.7 | 1.94 +/- 1.3 | 1.9 | 0.00013 | (30/38) | 79 |
| BCAM | basal cell adhesion molecule (Lutheran blood group) | 2.55 +/- 1.9 | 4.44 +/- 1.8 | 0.6 | 0.00014 | (7/38) | 18 |
| EHD3 | EH-domain containing 3 | 1.32 +/- 0.99 | 2.74 +/- 1.7 | 0.5 | 0.00014 | (6/38) | 16 |
| CALR | calreticulin | 8.9 +/- 3.4 | 5.01 +/- 2.9 | 1.8 | 0.00014 | (30/38) | 79 |
| MDH2 | malate dehydrogenase 2, NAD (mitochondrial) | 5.36 +/- 1.6 | 3.27 +/- 1.9 | 1.6 | 0.00014 | (29/38) | 76 |
| HSP90B1 | heat shock protein 90kDa beta (Grp94), member 1 | 10.1 +/- 5.3 | 5.21 +/- 4.4 | 1.9 | 0.00016 | (31/38) | 82 |
| EPB42 | erythrocyte membrane protein band 4.2 | 0.702 +/- 0.59 | 2.19 +/- 2.5 | 0.3 | 0.00017 | (8/38) | 21 |
| EIF4A1 | eukaryotic translation initiation factor 4A1 | 3.49 +/- 1.4 | 1.88 +/- 1.3 | 1.9 | 0.00019 | (30/38) | 79 |
| HSPA8 | heat shock 70kDa protein 8 | 6.78 +/- 2.9 | 4.44 +/- 2.3 | 1.5 | 0.00019 | (30/38) | 79 |
| EHD2 | EH-domain containing 2 | 5.46 +/- 4.1 | 10.2 +/- 4.4 | 0.5 | 0.00019 | (5/38) | 13 |
| EEF1G | eukaryotic translation elongation factor 1 gamma | 3.14 +/- 1.5 | 1.63 +/- 0.99 | 1.9 | 0.00019 | (31/38) | 82 |
| NANS | N-acetylneuraminic acid synthase | 1.64 +/- 1.1 | 0.61 +/- 0.55 | 2.7 | 0.00019 | (31/38) | 82 |
| UGGT1 | UDP-glucose glycoprotein glucosyltransferase 1 | 3.73 +/- 1.4 | 2.07 +/- 1.4 | 1.8 | 0.0002 | (29/38) | 76 |
| PGD | phosphogluconate dehydrogenase | 3.48 +/- 1.6 | 1.89 +/- 1.2 | 1.8 | 0.00022 | (30/38) | 79 |
| LDHA | lactate dehydrogenase A | 8.81 +/- 3.1 | 6.12 +/- 2.4 | 1.4 | 0.00022 | (30/38) | 79 |
| CFL1 | cofilin 1 (non-muscle) | 5.43 +/- 2.4 | 3.38 +/- 1.8 | 1.6 | 0.00022 | (26/38) | 68 |
| XRCC6 | X-ray repair complementing defective repair in Chinese hamster cells 6 | 4.66 +/- 1.7 | 2.8 +/- 1.7 | 1.7 | 0.00026 | (29/38) | 76 |
| GSTP1 | glutathione S-transferase pi 1 | 7.16 +/- 2.7 | 4.23 +/- 2.6 | 1.7 | 0.00026 | (27/38) | 71 |
| ERO1L | ERO1-like (S. cerevisiae) | 1.86 +/- 1.4 | 0.766 +/- 0.43 | 2.4 | 0.00026 | (33/38) | 87 |
| PDIA4 | protein disulfide isomerase family A, member 4 | 6.23 +/- 3.4 | 2.69 +/- 2.2 | 2.3 | 0.00027 | (31/38) | 82 |
| PGK1 | phosphoglycerate kinase 1 | 9.02 +/- 4.6 | 4.5 +/- 3.8 | 2 | 0.00027 | (30/38) | 79 |
| FH | fumarate hydratase | 2.22 +/- 1.2 | 1.02 +/- 0.68 | 2.2 | 0.00033 | (33/38) | 87 |
| STAT1 | signal transducer and activator of transcription 1, 91kDa | 4.66 +/- 2.6 | 2.82 +/- 1.7 | 1.6 | 0.00034 | (30/38) | 79 |
| OLFML1 | olfactomedin-like 1 | 3.03 +/- 2.4 | 4.92 +/- 1.9 | 0.6 | 0.00036 | (8/38) | 21 |
| TAGLN2 | transgelin 2 | 6.45 +/- 2.6 | 4.26 +/- 2.9 | 1.5 | 0.00037 | (25/38) | 66 |
| FUCA1 | fucosidase, alpha-L- 1, tissue | 1.44 +/- 1.5 | 0.523 +/- 0.28 | 2.8 | 0.00042 | (29/38) | 76 |
| PFN1 | profilin 1 | 4.74 +/- 2.2 | 2.91 +/- 2 | 1.6 | 0.00044 | (27/38) | 71 |
| MYO1C | myosin IC | 6.11 +/- 5.2 | 11.5 +/- 4.7 | 0.5 | 0.00044 | (9/38) | 24 |
| GPI | glucose-6-phosphate isomerase | 5.32 +/- 2.9 | 2.44 +/- 1.9 | 2.2 | 0.00044 | (29/38) | 76 |
| HBB | hemoglobin, beta | 56 +/- 44 | 113 +/- 83 | 0.5 | 0.00044 | (9/38) | 24 |
| YARS | tyrosyl-tRNA synthetase | 1.23 +/- 0.8 | 0.412 +/- 0.3 | 3 | 0.00048 | (32/38) | 84 |
| PPA1 | pyrophosphatase (inorganic) 1 | 2.38 +/- 1.2 | 1.16 +/- 0.75 | 2 | 0.00048 | (30/38) | 79 |
| COPA | coatomer protein complex, subunit alpha | 3.96 +/- 1.8 | 2.33 +/- 1.7 | 1.7 | 0.00051 | (29/38) | 76 |
| PSME1 | proteasome (prosome, macropain) activator subunit 1 (PA28 alpha) | 4.39 +/- 1.8 | 2.66 +/- 1.3 | 1.6 | 0.00051 | (29/38) | 76 |
| UBA1 | ubiquitin-like modifier activating enzyme 1 | 7.92 +/- 3.1 | 4.83 +/- 2.7 | 1.6 | 0.00056 | (29/38) | 76 |
| PLIN3 | perilipin 3 | 2.15 +/- 1.1 | 1.01 +/- 0.73 | 2.1 | 0.00057 | (31/38) | 82 |
| NME1-NME2 | NME1-NME2 readthrough | 3.73 +/- 2 | 2.17 +/- 1.4 | 1.7 | 0.00061 | (27/38) | 71 |
| ITGB1 | integrin, beta 1 (fibronectin receptor, beta polypeptide, antigen CD29 includes MDF2, MSK12) | 1.98 +/- 1.3 | 3.47 +/- 1.3 | 0.6 | 0.00063 | (8/38) | 21 |
| KRT19 | keratin 19 | 16.7 +/- 6.4 | 11.2 +/- 5.1 | 1.5 | 0.00066 | (28/38) | 74 |
| CAV1 | caveolin 1, caveolae protein, 22kDa | 1.82 +/- 1.2 | 3.26 +/- 1.7 | 0.6 | 0.00066 | (8/38) | 21 |
| HSPD1 | heat shock 60kDa protein 1 (chaperonin) | 12.6 +/- 6.3 | 6.71 +/- 4.3 | 1.9 | 0.00066 | (31/38) | 82 |
| HSPA4 | heat shock 70kDa protein 4 | 2 +/- 1.1 | 0.921 +/- 0.67 | 2.2 | 0.00066 | (31/38) | 82 |
| LAP3 | leucine aminopeptidase 3 | 3.76 +/- 2.1 | 2.2 +/- 1.7 | 1.7 | 0.00066 | (27/38) | 71 |
| COL6A2 | collagen, type VI, alpha 2 | 14 +/- 12 | 25.7 +/- 11 | 0.6 | 0.00067 | (11/38) | 29 |
| GLOD4 | glyoxalase domain containing 4 | 1.4 +/- 0.92 | 0.53 +/- 0.43 | 2.6 | 0.00067 | (30/38) | 79 |
| HBA1 | hemoglobin, alpha 1 | 42.7 +/- 35 | 88.3 +/- 74 | 0.5 | 0.0007 | (9/38) | 24 |
| S100A11 | S100 calcium binding protein A11 | 4.13 +/- 2 | 2.51 +/- 1.2 | 1.6 | 0.00075 | (28/38) | 74 |
| PDIA3 | protein disulfide isomerase family A, member 3 | 7.5 +/- 3.5 | 3.94 +/- 2.5 | 1.9 | 0.00076 | (30/38) | 79 |
| LONP1 | lon peptidase 1, mitochondrial | 1.66 +/- 1.1 | 0.708 +/- 0.52 | 2.4 | 0.00079 | (30/38) | 79 |
| HSPA5 | heat shock 70kDa protein 5 (glucose-regulated protein, 78kDa) | 10.1 +/- 4.5 | 5.97 +/- 4.1 | 1.7 | 0.00079 | (30/38) | 79 |
| FGA | fibrinogen alpha chain | 10.5 +/- 9.8 | 18.2 +/- 12 | 0.6 | 0.0009 | (9/38) | 24 |
| TXNDC5 | thioredoxin domain containing 5 (endoplasmic reticulum) | 3.73 +/- 1.8 | 2.22 +/- 1.6 | 1.7 | 0.0009 | (28/38) | 74 |
| HADH | hydroxyacyl-CoA dehydrogenase | 2.23 +/- 1.5 | 1.1 +/- 0.71 | 2 | 0.00093 | (28/38) | 74 |
| GOT1 | glutamic-oxaloacetic transaminase 1, soluble | 1.44 +/- 1.5 | 0.576 +/- 0.32 | 2.5 | 0.00096 | (30/38) | 79 |
| ANXA2 | annexin A2 | 7.93 +/- 5.5 | 13.8 +/- 5.8 | 0.6 | 0.00098 | (10/38) | 26 |
| GAPDH | glyceraldehyde-3-phosphate dehydrogenase | 22.9 +/- 16 | 12.2 +/- 8.5 | 1.9 | 0.00099 | (27/38) | 71 |
| CTSH | cathepsin H | 1.9 +/- 1.7 | 0.875 +/- 0.69 | 2.2 | 0.00098 | (27/38) | 71 |
| AGER | advanced glycosylation end product-specific receptor | 3.08 +/- 2.5 | 6 +/- 2.7 | 0.5 | 0.001 | (7/38) | 18 |
| RCN1 | reticulocalbin 1, EF-hand calcium binding domain | 1.15 +/- 0.84 | 0.4 +/- 0.34 | 2.9 | 0.0011 | (30/38) | 79 |
| ARHGDIA | Rho GDP dissociation inhibitor (GDI) alpha | 3 +/- 1.2 | 1.68 +/- 1.1 | 1.8 | 0.0011 | (29/38) | 76 |
| ARCN1 | archain 1 | 1.95 +/- 0.84 | 0.929 +/- 0.65 | 2.1 | 0.0012 | (31/38) | 82 |
| LAMC1 | laminin, gamma 1 (formerly LAMB2) | 9.18 +/- 7.2 | 15.6 +/- 6.3 | 0.6 | 0.0013 | (9/38) | 24 |
| PRELP | proline/arginine-rich end leucine-rich repeat protein | 8.28 +/- 6.2 | 14.3 +/- 6 | 0.6 | 0.0014 | (7/38) | 18 |
| DCN | decorin | 9.47 +/- 7.4 | 16.1 +/- 6.6 | 0.6 | 0.0015 | (11/38) | 29 |
| PARP1 | poly (ADP-ribose) polymerase 1 | 3.09 +/- 1.8 | 1.45 +/- 1.7 | 2.1 | 0.0015 | (28/38) | 74 |
| PSME2 | proteasome (prosome, macropain) activator subunit 2 (PA28 beta) | 3.13 +/- 1.4 | 1.81 +/- 0.98 | 1.7 | 0.0015 | (28/38) | 74 |
| VTN | vitronectin | 8.2 +/- 4.4 | 10.8 +/- 3.8 | 0.8 | 0.0015 | (11/38) | 29 |
| ARF4 | ADP-ribosylation factor 4 | 2.3 +/- 1 | 1.19 +/- 0.79 | 1.9 | 0.0015 | (29/38) | 76 |
| HSD17B10 | hydroxysteroid (17-beta) dehydrogenase 10 | 2.27 +/- 0.91 | 1.17 +/- 0.91 | 1.9 | 0.0015 | (27/38) | 71 |
| AOC3 | amine oxidase, copper containing 3 | 10.8 +/- 7 | 17 +/- 6 | 0.6 | 0.0016 | (7/38) | 18 |
| PA2G4 | proliferation-associated 2G4, 38kDa | 1.96 +/- 1 | 0.958 +/- 0.81 | 2 | 0.0017 | (31/38) | 82 |
| MTHFD1 | methylenetetrahydrofolate dehydrogenase (NADP+ dependent) 1, methenyltetrahydrofolate cyclohydrolase, formyltetrahydrofolate synthetase | 1.72 +/- 1 | 0.799 +/- 0.57 | 2.1 | 0.0018 | (31/38) | 82 |
| PEBP1 | phosphatidylethanolamine binding protein 1 | 2.87 +/- 1.3 | 1.63 +/- 1.4 | 1.8 | 0.0019 | (27/38) | 71 |
| COL6A1 | collagen, type VI, alpha 1 | 17.7 +/- 16 | 31.9 +/- 17 | 0.6 | 0.0019 | (11/38) | 29 |
| APRT | adenine phosphoribosyltransferase | 2.03 +/- 0.9 | 1.02 +/- 0.73 | 2 | 0.0019 | (28/38) | 74 |
| COL6A3 | collagen, type VI, alpha 3 | 46 +/- 34 | 77.9 +/- 34 | 0.6 | 0.0019 | (9/38) | 24 |
| LAMB2 | laminin, beta 2 (laminin S) | 11.9 +/- 9.7 | 21.1 +/- 9.2 | 0.6 | 0.0021 | (9/38) | 24 |
| ARF1 | ADP-ribosylation factor 1 | 4.08 +/- 1.8 | 2.59 +/- 1.4 | 1.6 | 0.0021 | (28/38) | 74 |
| AHNAK | AHNAK nucleoprotein | 29 +/- 15 | 44.7 +/- 19 | 0.6 | 0.0021 | (10/38) | 26 |
| LAMB3 | laminin, beta 3 | 4.85 +/- 4.2 | 8.87 +/- 4.5 | 0.6 | 0.0022 | (8/38) | 21 |
| P4HB | prolyl 4-hydroxylase, beta polypeptide | 9.12 +/- 4.2 | 4.32 +/- 3.1 | 2.1 | 0.0022 | (30/38) | 79 |
| G6PD | glucose-6-phosphate dehydrogenase | 2.36 +/- 1.9 | 1.27 +/- 1 | 1.9 | 0.0023 | (27/38) | 71 |
| HNRNPA2B1 | heterogeneous nuclear ribonucleoprotein A2/B1 | 6.75 +/- 2.8 | 4.79 +/- 2.5 | 1.4 | 0.0023 | (26/38) | 68 |
| ARHGAP1 | Rho GTPase activating protein 1 | 2.86 +/- 1.2 | 1.65 +/- 0.89 | 1.7 | 0.0023 | (29/38) | 76 |
| RPL4 | ribosomal protein L4 | 2.62 +/- 1.1 | 1.47 +/- 1.3 | 1.8 | 0.0023 | (29/38) | 76 |
| PPA2 | pyrophosphatase (inorganic) 2 | 1.93 +/- 1.1 | 0.963 +/- 0.72 | 2 | 0.0023 | (26/38) | 68 |
| COL6A6 | collagen, type VI, alpha 6 | 22.7 +/- 17 | 37.3 +/- 14 | 0.6 | 0.0025 | (9/38) | 24 |
| DDX39B | DEAD (Asp-Glu-Ala-Asp) box polypeptide 39B | 2.18 +/- 0.9 | 1.15 +/- 0.77 | 1.9 | 0.0025 | (30/38) | 79 |
| PDXK | pyridoxal (pyridoxine, vitamin B6) kinase | 1.93 +/- 1.1 | 0.973 +/- 0.77 | 2 | 0.0025 | (26/38) | 68 |
| KRT8 | keratin 8 | 15.4 +/- 8.5 | 9.78 +/- 5.5 | 1.6 | 0.0025 | (26/38) | 68 |
| EEF1A2 | eukaryotic translation elongation factor 1 alpha 2 | 4.53 +/- 1.6 | 2.98 +/- 1.6 | 1.5 | 0.0025 | (28/38) | 74 |
| EIF5A | eukaryotic translation initiation factor 5A | 3.23 +/- 1.3 | 1.94 +/- 1.2 | 1.7 | 0.0025 | (29/38) | 76 |
| NME1 | NME/NM23 nucleoside diphosphate kinase 1 | 2 +/- 1 | 1.02 +/- 0.63 | 2 | 0.0025 | (30/38) | 79 |
| HSPG2 | heparan sulfate proteoglycan 2 | 26.1 +/- 17 | 42.1 +/- 16 | 0.6 | 0.0025 | (12/38) | 32 |
| LMNB1 | lamin B1 | 4.56 +/- 1.8 | 3.01 +/- 2.4 | 1.5 | 0.0026 | (30/38) | 79 |
| NPEPPS | aminopeptidase puromycin sensitive | 3.68 +/- 1.7 | 2.31 +/- 1.4 | 1.6 | 0.0025 | (29/38) | 76 |
| ARF3 | ADP-ribosylation factor 3 | 4.03 +/- 1.8 | 2.58 +/- 1.4 | 1.6 | 0.0025 | (28/38) | 74 |
| FGB | fibrinogen beta chain | 11.6 +/- 11 | 20.5 +/- 14 | 0.6 | 0.0026 | (12/38) | 32 |
| FGG | fibrinogen gamma chain | 8.3 +/- 6.9 | 15 +/- 11 | 0.6 | 0.0026 | (13/38) | 34 |
| RPS3 | ribosomal protein S3 | 2.77 +/- 1.1 | 1.6 +/- 1.1 | 1.7 | 0.0026 | (30/38) | 79 |
| KRT18 | keratin 18 | 13.7 +/- 6.4 | 9.21 +/- 4.9 | 1.5 | 0.0026 | (27/38) | 71 |
| NID1 | nidogen 1 | 5.15 +/- 4.1 | 8.48 +/- 3.4 | 0.6 | 0.0027 | (9/38) | 24 |
| TPD52 | tumor protein D52 | 0.942 +/- 0.64 | 0.324 +/- 0.33 | 2.9 | 0.0027 | (29/38) | 76 |
| HNRNPK | heterogeneous nuclear ribonucleoprotein K | 3.93 +/- 1.7 | 2.51 +/- 1.5 | 1.6 | 0.0027 | (30/38) | 79 |
| ECHS1 | enoyl CoA hydratase, short chain, 1, mitochondrial | 2.14 +/- 0.81 | 1.13 +/- 0.83 | 1.9 | 0.0028 | (29/38) | 76 |
| DPYSL2 | dihydropyrimidinase-like 2 | 3.43 +/- 2 | 5.06 +/- 1.9 | 0.7 | 0.0028 | (8/38) | 21 |
| CORO1A | coronin, actin binding protein, 1A | 3.26 +/- 1.3 | 1.99 +/- 1.3 | 1.6 | 0.0029 | (29/38) | 76 |
| SDHA | succinate dehydrogenase complex, subunit A, flavoprotein (Fp) | 1.74 +/- 0.92 | 0.854 +/- 0.66 | 2 | 0.0029 | (32/38) | 84 |
| ARPC2 | actin related protein 2/3 complex, subunit 2, 34kDa | 3.21 +/- 1.6 | 1.96 +/- 1.3 | 1.6 | 0.003 | (30/38) | 79 |
| MYH10 | myosin, heavy chain 10, non-muscle | 18.9 +/- 12 | 30.9 +/- 12 | 0.6 | 0.003 | (9/38) | 24 |
| TPSAB1 | tryptase alpha/beta 1 | 6.32 +/- 3.5 | 8.45 +/- 3.3 | 0.8 | 0.003 | (11/38) | 29 |
| HSPB1 | heat shock 27kDa protein 1 | 6.22 +/- 2.6 | 4.41 +/- 2.1 | 1.4 | 0.0031 | (27/38) | 71 |
| WARS | tryptophanyl-tRNA synthetase | 4.34 +/- 2.4 | 2.86 +/- 2 | 1.5 | 0.0032 | (24/38) | 63 |
| SET | SET nuclear proto-oncogene | 1.33 +/- 0.78 | 0.578 +/- 0.6 | 2.3 | 0.0033 | (31/38) | 82 |
| TPSB2 | tryptase beta 2 (gene/pseudogene) | 6.3 +/- 3.5 | 8.41 +/- 3.2 | 0.8 | 0.0033 | (11/38) | 29 |
| GDI1 | GDP dissociation inhibitor 1 | 3.4 +/- 1.2 | 2.12 +/- 1.1 | 1.6 | 0.0033 | (29/38) | 76 |
| SERPINH1 | serpin peptidase inhibitor, clade H (heat shock protein 47), member 1, (collagen binding protein 1) | 4.23 +/- 1.9 | 2.79 +/- 1.7 | 1.5 | 0.0034 | (26/38) | 68 |
| TXNRD1 | thioredoxin reductase 1 | 2.18 +/- 1.4 | 1.19 +/- 0.77 | 1.8 | 0.0036 | (30/38) | 79 |
| COPG1 | coatomer protein complex, subunit gamma 1 | 2.67 +/- 1.4 | 1.56 +/- 1 | 1.7 | 0.0036 | (29/38) | 76 |
| TMSB4X | thymosin beta 4, X-linked | 4.75 +/- 2 | 3.22 +/- 2.4 | 1.5 | 0.0036 | (26/38) | 68 |
| BGN | biglycan | 13.7 +/- 9.4 | 21.3 +/- 9 | 0.6 | 0.0038 | (10/38) | 26 |
| TINAGL1 | tubulointerstitial nephritis antigen-like 1 | 1.53 +/- 1.1 | 2.62 +/- 1.1 | 0.6 | 0.0038 | (11/38) | 29 |
| CALU | calumenin | 1.69 +/- 1.1 | 0.84 +/- 0.71 | 2 | 0.0039 | (30/38) | 79 |
| YWHAZ | tyrosine 3-monooxygenase/tryptophan 5-monooxygenase activation protein, zeta | 5.94 +/- 2 | 4.23 +/- 2 | 1.4 | 0.004 | (25/38) | 66 |
| APOH | apolipoprotein H (beta-2-glycoprotein I) | 3.15 +/- 1.8 | 4.65 +/- 2.3 | 0.7 | 0.004 | (12/38) | 32 |
| ALDH6A1 | aldehyde dehydrogenase 6 family, member A1 | 1.23 +/- 1.1 | 0.531 +/- 0.46 | 2.3 | 0.0042 | (29/38) | 76 |
| ACTN4 | actinin, alpha 4 | 5.38 +/- 2.3 | 7.29 +/- 3.3 | 0.7 | 0.0042 | (13/38) | 34 |
| HSP90AA1 | heat shock protein 90kDa alpha (cytosolic), class A member 1 | 10.4 +/- 4 | 6.61 +/- 3.4 | 1.6 | 0.0042 | (33/38) | 87 |
| TPI1 | triosephosphate isomerase 1 | 7.64 +/- 3.1 | 4.36 +/- 2.9 | 1.8 | 0.0042 | (26/38) | 68 |
| LMAN1 | lectin, mannose-binding, 1 | 2.28 +/- 0.99 | 1.28 +/- 1.2 | 1.8 | 0.0044 | (33/38) | 87 |
| FBP1 | fructose-1,6-bisphosphatase 1 | 2.78 +/- 1.7 | 1.66 +/- 1.2 | 1.7 | 0.0046 | (27/38) | 71 |
| ALDH7A1 | aldehyde dehydrogenase 7 family, member A1 | 1.38 +/- 1.1 | 0.637 +/- 0.48 | 2.2 | 0.0046 | (29/38) | 76 |
| COTL1 | coactosin-like F-actin binding protein 1 | 1.74 +/- 0.82 | 0.892 +/- 0.71 | 2 | 0.0047 | (32/38) | 84 |
| RPS7 | ribosomal protein S7 | 2.92 +/- 1.1 | 1.77 +/- 1 | 1.6 | 0.0045 | (30/38) | 79 |
| SF3B1 | splicing factor 3b, subunit 1, 155kDa | 2.1 +/- 0.86 | 1.15 +/- 0.93 | 1.8 | 0.0049 | (31/38) | 82 |
| NPM1 | nucleophosmin (nucleolar phosphoprotein B23, numatrin) | 4.67 +/- 1.8 | 3.19 +/- 1.5 | 1.5 | 0.0048 | (29/38) | 76 |
| ETFA | electron-transfer-flavoprotein, alpha polypeptide | 1.91 +/- 0.84 | 1.01 +/- 0.63 | 1.9 | 0.0048 | (27/38) | 71 |
| CNPY2 | canopy FGF signaling regulator 2 | 1.35 +/- 0.69 | 0.619 +/- 0.53 | 2.2 | 0.0048 | (31/38) | 82 |
| BLVRA | biliverdin reductase A | 1.37 +/- 0.8 | 0.638 +/- 0.41 | 2.1 | 0.0053 | (32/38) | 84 |
| TALDO1 | transaldolase 1 | 2.61 +/- 1.4 | 1.56 +/- 1.3 | 1.7 | 0.0056 | (24/38) | 63 |
| SHMT2 | serine hydroxymethyltransferase 2 (mitochondrial) | 1.16 +/- 0.94 | 0.499 +/- 0.54 | 2.3 | 0.0056 | (33/38) | 87 |
| YWHAH | tyrosine 3-monooxygenase/tryptophan 5-monooxygenase activation protein, eta | 2.81 +/- 1.2 | 1.71 +/- 0.98 | 1.6 | 0.0057 | (27/38) | 71 |
| PPP1CA | protein phosphatase 1, catalytic subunit, alpha isozyme | 1.69 +/- 0.69 | 0.872 +/- 0.58 | 1.9 | 0.0057 | (31/38) | 82 |
| ARF5 | ADP-ribosylation factor 5 | 2.14 +/- 0.87 | 1.2 +/- 0.73 | 1.8 | 0.0061 | (28/38) | 74 |
| RPS4X | ribosomal protein S4, X-linked | 3.37 +/- 1.3 | 2.17 +/- 1.2 | 1.6 | 0.0064 | (29/38) | 76 |
| RPS8 | ribosomal protein S8 | 1.79 +/- 0.78 | 0.948 +/- 0.94 | 1.9 | 0.0064 | (28/38) | 74 |
| LCP1 | lymphocyte cytosolic protein 1 (L-plastin) | 10.7 +/- 3.6 | 8.45 +/- 3.6 | 1.3 | 0.007 | (27/38) | 71 |
| GBP1 | guanylate binding protein 1, interferon-inducible | 1.63 +/- 1.1 | 0.839 +/- 0.46 | 1.9 | 0.0069 | (32/38) | 84 |
| KRT15 | keratin 15 | 1.74 +/- 1.2 | 0.922 +/- 0.57 | 1.9 | 0.007 | (28/38) | 74 |
| EIF4A2 | eukaryotic translation initiation factor 4A2 | 2.67 +/- 0.95 | 1.62 +/- 0.91 | 1.6 | 0.0069 | (30/38) | 79 |
| RRBP1 | ribosome binding protein 1 | 5.23 +/- 2.7 | 2.67 +/- 2.5 | 2 | 0.0071 | (31/38) | 82 |
| ANXA4 | annexin A4 | 2.6 +/- 1.7 | 1.58 +/- 0.88 | 1.6 | 0.0072 | (26/38) | 68 |
| PRDX4 | peroxiredoxin 4 | 2.38 +/- 1.2 | 1.41 +/- 0.95 | 1.7 | 0.0077 | (30/38) | 79 |
| RAN | RAN, member RAS oncogene family | 1.71 +/- 0.81 | 0.905 +/- 0.71 | 1.9 | 0.0077 | (33/38) | 87 |
| ATP5B | ATP synthase, H+ transporting, mitochondrial F1 complex, beta polypeptide | 8.45 +/- 3.9 | 6.51 +/- 2.7 | 1.3 | 0.0078 | (26/38) | 68 |
| DDX3X | DEAD (Asp-Glu-Ala-Asp) box helicase 3, X-linked | 1.79 +/- 0.85 | 0.969 +/- 0.61 | 1.8 | 0.0078 | (28/38) | 74 |
| CS | citrate synthase | 2.33 +/- 1.3 | 1.37 +/- 0.78 | 1.7 | 0.0079 | (28/38) | 74 |
| ERP44 | endoplasmic reticulum protein 44 | 1.44 +/- 0.83 | 0.71 +/- 0.52 | 2 | 0.0077 | (31/38) | 82 |
| CRYZ | crystallin, zeta (quinone reductase) | 1.52 +/- 0.96 | 0.777 +/- 0.55 | 2 | 0.0079 | (29/38) | 76 |
| CD36 | CD36 molecule (thrombospondin receptor) | 0.83 +/- 0.93 | 1.6 +/- 0.97 | 0.5 | 0.0079 | (10/38) | 26 |
| MYOF | myoferlin | 5.74 +/- 3.5 | 4.18 +/- 2.2 | 1.4 | 0.0084 | (26/38) | 68 |
| STOML2 | stomatin (EPB72)-like 2 | 1.38 +/- 0.57 | 0.675 +/- 0.39 | 2 | 0.0084 | (32/38) | 84 |
| ETFB | electron-transfer-flavoprotein, beta polypeptide | 1.08 +/- 0.49 | 0.473 +/- 0.43 | 2.3 | 0.0083 | (31/38) | 82 |
| PSMA4 | proteasome (prosome, macropain) subunit, alpha type, 4 | 1.03 +/- 0.55 | 0.442 +/- 0.38 | 2.3 | 0.0084 | (32/38) | 84 |
| PLG | plasminogen | 1.43 +/- 1.2 | 2.38 +/- 1.6 | 0.6 | 0.009 | (13/38) | 34 |
| SSB | Sjogren syndrome antigen B (autoantigen La) | 2.12 +/- 1.3 | 1.23 +/- 0.97 | 1.7 | 0.0089 | (28/38) | 74 |
| RPS12 | ribosomal protein S12 | 3.42 +/- 1.2 | 2.26 +/- 1.1 | 1.5 | 0.0094 | (30/38) | 79 |
| PRDX2 | peroxiredoxin 2 | 3.58 +/- 1.7 | 5.01 +/- 2.4 | 0.7 | 0.0096 | (16/38) | 42 |
| ME2 | malic enzyme 2, NAD(+)-dependent, mitochondrial | 0.96 +/- 0.77 | 0.4 +/- 0.32 | 2.4 | 0.0095 | (33/38) | 87 |
| CA4 | carbonic anhydrase IV | 1.04 +/- 0.92 | 1.86 +/- 1.2 | 0.6 | 0.0098 | (11/38) | 29 |
| CAP1 | CAP, adenylate cyclase-associated protein 1 (yeast) | 6.2 +/- 2 | 4.61 +/- 2 | 1.3 | 0.01 | (24/38) | 63 |
| PYGL | phosphorylase, glycogen, liver | 1.92 +/- 1.1 | 1.09 +/- 0.61 | 1.8 | 0.01 | (28/38) | 74 |
| EFTUD2 | elongation factor Tu GTP binding domain containing 2 | 2.14 +/- 0.82 | 1.26 +/- 0.72 | 1.7 | 0.01 | (31/38) | 82 |
| LMNA | lamin A/C | 9.07 +/- 3.8 | 11.3 +/- 5.1 | 0.8 | 0.01 | (15/38) | 39 |
| DARS | aspartyl-tRNA synthetase | 1.27 +/- 0.7 | 0.616 +/- 0.44 | 2.1 | 0.01 | (31/38) | 82 |
| EIF2S3 | eukaryotic translation initiation factor 2, subunit 3 gamma, 52kDa | 1.32 +/- 0.66 | 0.653 +/- 0.54 | 2 | 0.01 | (31/38) | 82 |
| CKAP4 | cytoskeleton-associated protein 4 | 3.56 +/- 1.4 | 2.39 +/- 1.9 | 1.5 | 0.011 | (27/38) | 71 |
| RPL7A | ribosomal protein L7a | 1.85 +/- 1.1 | 1.04 +/- 0.83 | 1.8 | 0.011 | (28/38) | 74 |
| NSFL1C | NSFL1 (p97) cofactor (p47) | 0.769 +/- 0.57 | 0.287 +/- 0.18 | 2.7 | 0.011 | (31/38) | 82 |
| C4B | complement component 4B (Chido blood group) | 5.65 +/- 4.4 | 8.69 +/- 5.3 | 0.6 | 0.011 | (11/38) | 29 |
| EHD4 | EH-domain containing 4 | 1.04 +/- 0.84 | 1.84 +/- 1.1 | 0.6 | 0.011 | (10/38) | 26 |
| SLC25A5 | solute carrier family 25 (mitochondrial carrier; adenine nucleotide translocator), member 5 | 2.33 +/- 1 | 1.42 +/- 0.9 | 1.6 | 0.011 | (28/38) | 74 |
| NUCB1 | nucleobindin 1 | 2.01 +/- 0.74 | 1.17 +/- 0.77 | 1.7 | 0.011 | (30/38) | 79 |
| CAPN2 | calpain 2, (m/II) large subunit | 3.66 +/- 1.8 | 2.49 +/- 1.3 | 1.5 | 0.012 | (27/38) | 71 |
| SCP2 | sterol carrier protein 2 | 0.845 +/- 0.57 | 0.341 +/- 0.28 | 2.5 | 0.012 | (30/38) | 79 |
| NOP56 | NOP56 ribonucleoprotein | 1.29 +/- 0.85 | 0.642 +/- 0.81 | 2 | 0.013 | (31/38) | 82 |
| PSMC4 | proteasome (prosome, macropain) 26S subunit, ATPase, 4 | 1.73 +/- 0.84 | 0.966 +/- 0.68 | 1.8 | 0.013 | (29/38) | 76 |
| C4A | complement component 4A (Rodgers blood group) | 5.68 +/- 4.3 | 8.58 +/- 5.3 | 0.7 | 0.013 | (12/38) | 32 |
| USP14 | ubiquitin specific peptidase 14 (tRNA-guanine transglycosylase) | 0.868 +/- 0.51 | 0.361 +/- 0.25 | 2.4 | 0.014 | (32/38) | 84 |
| PPIB | peptidylprolyl isomerase B (cyclophilin B) | 4.78 +/- 1.3 | 3.46 +/- 1.7 | 1.4 | 0.014 | (31/38) | 82 |
| UGP2 | UDP-glucose pyrophosphorylase 2 | 1.87 +/- 0.79 | 1.08 +/- 0.64 | 1.7 | 0.014 | (30/38) | 79 |
| NAGK | N-acetylglucosamine kinase | 0.862 +/- 0.53 | 0.36 +/- 0.32 | 2.4 | 0.014 | (29/38) | 76 |
| MAPRE1 | microtubule-associated protein, RP/EB family, member 1 | 1.44 +/- 0.69 | 0.764 +/- 0.51 | 1.9 | 0.014 | (27/38) | 71 |
| HNRNPM | heterogeneous nuclear ribonucleoprotein M | 3.63 +/- 2 | 2.5 +/- 1.7 | 1.4 | 0.015 | (25/38) | 66 |
| RPL6 | ribosomal protein L6 | 3.58 +/- 1.4 | 2.46 +/- 1.6 | 1.5 | 0.015 | (28/38) | 74 |
| CBR1 | carbonyl reductase 1 | 3.08 +/- 1.4 | 2.04 +/- 2 | 1.5 | 0.016 | (26/38) | 68 |
| C3 | complement component 3 | 15.2 +/- 9.9 | 22.8 +/- 12 | 0.7 | 0.017 | (12/38) | 32 |
| HSP90AB1 | heat shock protein 90kDa alpha (cytosolic), class B member 1 | 10.2 +/- 4 | 6.75 +/- 3.6 | 1.5 | 0.017 | (30/38) | 79 |
| TUBB4B | tubulin, beta 4B class IVb | 5.88 +/- 3 | 7.54 +/- 3 | 0.8 | 0.017 | (12/38) | 32 |
| TRIM28 | tripartite motif containing 28 | 1.79 +/- 1 | 1.04 +/- 0.66 | 1.7 | 0.017 | (28/38) | 74 |
| SAR1A | secretion associated, Ras related GTPase 1A | 1.04 +/- 0.45 | 0.486 +/- 0.37 | 2.1 | 0.017 | (30/38) | 79 |
| ESD | esterase D | 1.68 +/- 0.93 | 0.954 +/- 0.88 | 1.8 | 0.018 | (28/38) | 74 |
| EZR | ezrin | 5.12 +/- 2.2 | 3.79 +/- 1.9 | 1.4 | 0.018 | (29/38) | 76 |
| RCC1 | regulator of chromosome condensation 1 | 0.847 +/- 0.75 | 0.363 +/- 0.36 | 2.3 | 0.018 | (27/38) | 71 |
| MVP | major vault protein | 7.85 +/- 3.9 | 4.61 +/- 2.7 | 1.7 | 0.018 | (28/38) | 74 |
| CLIC1 | chloride intracellular channel 1 | 2.98 +/- 1.3 | 1.99 +/- 1 | 1.5 | 0.018 | (26/38) | 68 |
| CANX | calnexin | 3.52 +/- 1.5 | 2.44 +/- 1.3 | 1.4 | 0.019 | (29/38) | 76 |
| TLN1 | talin 1 | 9.92 +/- 5.1 | 16.2 +/- 5.5 | 0.6 | 0.019 | (9/38) | 24 |
| TUBB | tubulin, beta class I | 5.02 +/- 2.6 | 6.53 +/- 2.7 | 0.8 | 0.019 | (12/38) | 32 |
| RPLP0 | ribosomal protein, large, P0 | 2.26 +/- 0.87 | 1.42 +/- 0.8 | 1.6 | 0.019 | (32/38) | 84 |
| ANXA3 | annexin A3 | 2.7 +/- 1.7 | 3.82 +/- 1.8 | 0.7 | 0.02 | (11/38) | 29 |
| PRDX3 | peroxiredoxin 3 | 2.43 +/- 0.76 | 1.55 +/- 0.9 | 1.6 | 0.02 | (30/38) | 79 |
| A2M | alpha-2-macroglobulin | 14.9 +/- 6.1 | 20.3 +/- 10 | 0.7 | 0.02 | (15/38) | 39 |
| COPE | coatomer protein complex, subunit epsilon | 1.21 +/- 0.64 | 0.625 +/- 0.6 | 1.9 | 0.02 | (27/38) | 71 |
| SH3BGRL | SH3 domain binding glutamate-rich protein like | 1.4 +/- 0.83 | 0.76 +/- 0.67 | 1.8 | 0.02 | (30/38) | 79 |
| HNRNPL | heterogeneous nuclear ribonucleoprotein L | 2.4 +/- 1.1 | 1.54 +/- 1 | 1.6 | 0.021 | (26/38) | 68 |
| ILF2 | interleukin enhancer binding factor 2 | 2.48 +/- 0.84 | 1.6 +/- 0.82 | 1.6 | 0.021 | (30/38) | 79 |
| ACTR3 | ARP3 actin-related protein 3 homolog (yeast) | 3.67 +/- 1.1 | 2.58 +/- 0.97 | 1.4 | 0.021 | (30/38) | 79 |
| PSMA7 | proteasome (prosome, macropain) subunit, alpha type, 7 | 1.54 +/- 0.85 | 0.871 +/- 0.57 | 1.8 | 0.021 | (29/38) | 76 |
| PIGR | polymeric immunoglobulin receptor | 3.65 +/- 4.4 | 2.13 +/- 1.5 | 1.7 | 0.021 | (22/38) | 58 |
| PSMB2 | proteasome (prosome, macropain) subunit, beta type, 2 | 1.14 +/- 0.56 | 0.578 +/- 0.33 | 2 | 0.021 | (31/38) | 82 |
| AKR1C1 | aldo-keto reductase family 1, member C1 | 2.18 +/- 2.4 | 1.09 +/- 0.96 | 2 | 0.021 | (27/38) | 71 |
| BASP1 | brain abundant, membrane attached signal protein 1 | 1.36 +/- 0.77 | 0.735 +/- 0.69 | 1.8 | 0.022 | (29/38) | 76 |
| GSTO1 | glutathione S-transferase omega 1 | 1.73 +/- 0.87 | 1.02 +/- 0.67 | 1.7 | 0.022 | (27/38) | 71 |
| AK3 | adenylate kinase 3 | 0.919 +/- 0.52 | 0.425 +/- 0.32 | 2.2 | 0.022 | (31/38) | 82 |
| ISOC2 | isochorismatase domain containing 2 | 0.759 +/- 0.49 | 0.319 +/- 0.22 | 2.4 | 0.022 | (32/38) | 84 |
| PPP1CB | protein phosphatase 1, catalytic subunit, beta isozyme | 1.69 +/- 0.65 | 0.995 +/- 0.59 | 1.7 | 0.024 | (33/38) | 87 |
| YWHAE | tyrosine 3-monooxygenase/tryptophan 5-monooxygenase activation protein, epsilon | 4.11 +/- 1.3 | 2.97 +/- 1.2 | 1.4 | 0.024 | (29/38) | 76 |
| PSMB1 | proteasome (prosome, macropain) subunit, beta type, 1 | 1.23 +/- 0.78 | 0.652 +/- 0.4 | 1.9 | 0.024 | (30/38) | 79 |
| COPB2 | coatomer protein complex, subunit beta 2 (beta prime) | 2.37 +/- 1 | 1.53 +/- 1.1 | 1.6 | 0.024 | (29/38) | 76 |
| RHOA | ras homolog family member A | 2.09 +/- 1.1 | 1.31 +/- 0.83 | 1.6 | 0.024 | (29/38) | 76 |
| COPB1 | coatomer protein complex, subunit beta 1 | 2.51 +/- 1.2 | 1.65 +/- 1.2 | 1.5 | 0.025 | (29/38) | 76 |
| FLNA | filamin A, alpha | 29 +/- 16 | 38.7 +/- 10 | 0.8 | 0.026 | (9/38) | 24 |
| LSP1 | lymphocyte-specific protein 1 | 1.08 +/- 0.59 | 0.551 +/- 0.55 | 2 | 0.026 | (30/38) | 79 |
| ATP6V1B2 | ATPase, H+ transporting, lysosomal 56/58kDa, V1 subunit B2 | 2.06 +/- 0.88 | 1.29 +/- 0.88 | 1.6 | 0.026 | (30/38) | 79 |
| MYL6 | myosin, light chain 6, alkali, smooth muscle and non-muscle | 2.51 +/- 1.5 | 3.54 +/- 1.8 | 0.7 | 0.026 | (11/38) | 29 |
| RNPEP | arginyl aminopeptidase (aminopeptidase B) | 1.8 +/- 1.1 | 1.09 +/- 0.68 | 1.6 | 0.028 | (25/38) | 66 |
| SUSD2 | sushi domain containing 2 | 2.35 +/- 2 | 3.34 +/- 1.8 | 0.7 | 0.028 | (10/38) | 26 |
| CD74 | CD74 molecule, major histocompatibility complex, class II invariant chain | 2.67 +/- 1.5 | 1.79 +/- 0.99 | 1.5 | 0.027 | (29/38) | 76 |
| SFXN1 | sideroflexin 1 | 0.816 +/- 0.52 | 0.368 +/- 0.35 | 2.2 | 0.028 | (30/38) | 79 |
| ALDH2 | aldehyde dehydrogenase 2 family (mitochondrial) | 5.32 +/- 2.3 | 4.06 +/- 2.1 | 1.3 | 0.03 | (25/38) | 66 |
| ALDOA | aldolase A, fructose-bisphosphate | 10.4 +/- 5.4 | 5.29 +/- 2.9 | 2 | 0.03 | (28/38) | 74 |
| NAPRT | nicotinate phosphoribosyltransferase | 1.19 +/- 0.81 | 0.637 +/- 0.51 | 1.9 | 0.028 | (28/38) | 74 |
| DBNL | drebrin-like | 1.26 +/- 0.63 | 0.69 +/- 0.45 | 1.8 | 0.03 | (29/38) | 76 |
| FKBP2 | FK506 binding protein 2, 13kDa | 0.568 +/- 0.4 | 0.212 +/- 0.29 | 2.7 | 0.03 | (30/38) | 79 |
| ADH1B | alcohol dehydrogenase 1B (class I), beta polypeptide | 6.33 +/- 4.6 | 8.81 +/- 3.8 | 0.7 | 0.031 | (12/38) | 32 |
| PSMD2 | proteasome (prosome, macropain) 26S subunit, non-ATPase, 2 | 2.18 +/- 0.78 | 1.41 +/- 0.62 | 1.5 | 0.032 | (32/38) | 84 |
| LUM | lumican | 19.8 +/- 16 | 28.8 +/- 14 | 0.7 | 0.03 | (9/38) | 24 |
| LIMA1 | LIM domain and actin binding 1 | 0.946 +/- 0.8 | 0.468 +/- 0.31 | 2 | 0.032 | (31/38) | 82 |
| YWHAQ | tyrosine 3-monooxygenase/tryptophan 5-monooxygenase activation protein, theta | 2.64 +/- 0.95 | 1.79 +/- 0.9 | 1.5 | 0.031 | (26/38) | 68 |
| SERPINB1 | serpin peptidase inhibitor, clade B (ovalbumin), member 1 | 2.26 +/- 1.3 | 1.48 +/- 0.93 | 1.5 | 0.031 | (29/38) | 76 |
| ASPN | asporin | 2.12 +/- 1.1 | 3.05 +/- 1.7 | 0.7 | 0.031 | (10/38) | 26 |
| PSMA1 | proteasome (prosome, macropain) subunit, alpha type, 1 | 1.28 +/- 0.76 | 0.704 +/- 0.48 | 1.8 | 0.03 | (28/38) | 74 |
| NUCB2 | nucleobindin 2 | 0.782 +/- 0.51 | 0.352 +/- 0.43 | 2.2 | 0.03 | (30/38) | 79 |
| ARPC3 | actin related protein 2/3 complex, subunit 3, 21kDa | 1.52 +/- 0.81 | 0.888 +/- 0.59 | 1.7 | 0.03 | (26/38) | 68 |
| PON1 | paraoxonase 1 | 0.384 +/- 0.5 | 0.83 +/- 1 | 0.5 | 0.03 | (16/38) | 42 |
| RPS17 | ribosomal protein S17 | 1.86 +/- 0.74 | 1.15 +/- 0.65 | 1.6 | 0.03 | (31/38) | 82 |
| EIF1 | eukaryotic translation initiation factor 1 | 0.59 +/- 0.43 | 0.229 +/- 0.21 | 2.6 | 0.032 | (31/38) | 82 |
| IQGAP1 | IQ motif containing GTPase activating protein 1 | 8.9 +/- 3.8 | 7.26 +/- 3.4 | 1.2 | 0.033 | (22/38) | 58 |
| ACADVL | acyl-CoA dehydrogenase, very long chain | 2.07 +/- 1.1 | 1.33 +/- 0.64 | 1.6 | 0.032 | (23/38) | 61 |
| KRT2 | keratin 2 | 1.17 +/- 0.67 | 1.86 +/- 1.9 | 0.6 | 0.033 | (12/38) | 32 |
| FSCN1 | fascin actin-bundling protein 1 | 1.78 +/- 1.2 | 1.1 +/- 0.76 | 1.6 | 0.033 | (26/38) | 68 |
| CAPG | capping protein (actin filament), gelsolin-like | 5.19 +/- 2 | 3.97 +/- 2.3 | 1.3 | 0.032 | (25/38) | 66 |
| TWF2 | twinfilin actin-binding protein 2 | 1.11 +/- 0.61 | 0.59 +/- 0.38 | 1.9 | 0.032 | (29/38) | 76 |
| DDAH1 | dimethylarginine dimethylaminohydrolase 1 | 0.909 +/- 0.75 | 0.444 +/- 0.27 | 2 | 0.032 | (28/38) | 74 |
| MPST | mercaptopyruvate sulfurtransferase | 0.768 +/- 0.58 | 0.348 +/- 0.26 | 2.2 | 0.032 | (29/38) | 76 |
| SFPQ | splicing factor proline/glutamine-rich | 2.82 +/- 1 | 1.95 +/- 1.2 | 1.4 | 0.034 | (29/38) | 76 |
| SERPINC1 | serpin peptidase inhibitor, clade C (antithrombin), member 1 | 2.09 +/- 1.6 | 2.98 +/- 1.9 | 0.7 | 0.035 | (13/38) | 34 |
| TIMP3 | TIMP metallopeptidase inhibitor 3 | 6.17 +/- 3.3 | 7.65 +/- 3.6 | 0.8 | 0.035 | (11/38) | 29 |
| ALDH1B1 | aldehyde dehydrogenase 1 family, member B1 | 1.17 +/- 0.78 | 0.637 +/- 0.34 | 1.8 | 0.033 | (34/38) | 89 |
| VAPA | VAMP (vesicle-associated membrane protein)-associated protein A, 33kDa | 1.58 +/- 0.79 | 0.953 +/- 0.59 | 1.7 | 0.035 | (27/38) | 71 |
| SEC23A | Sec23 homolog A (S. cerevisiae) | 1.44 +/- 0.93 | 0.845 +/- 0.66 | 1.7 | 0.036 | (27/38) | 71 |
| GPX1 | glutathione peroxidase 1 | 1.93 +/- 0.88 | 1.23 +/- 0.75 | 1.6 | 0.036 | (29/38) | 76 |
| RHOC | ras homolog family member C | 1.37 +/- 0.73 | 0.791 +/- 0.55 | 1.7 | 0.036 | (30/38) | 79 |
| TAGLN | transgelin | 6.62 +/- 2.9 | 5.28 +/- 2.9 | 1.2 | 0.039 | (22/38) | 58 |
| CA1 | carbonic anhydrase I | 5.84 +/- 4 | 8.52 +/- 6.1 | 0.7 | 0.039 | (14/38) | 37 |
| NPC2 | Niemann-Pick disease, type C2 | 1.59 +/- 0.98 | 0.972 +/- 0.82 | 1.6 | 0.039 | (26/38) | 68 |
| GNPDA1 | glucosamine-6-phosphate deaminase 1 | 0.584 +/- 0.39 | 0.236 +/- 0.24 | 2.5 | 0.038 | (33/38) | 87 |
| DBI | diazepam binding inhibitor (GABA receptor modulator, acyl-CoA binding protein) | 1.57 +/- 0.86 | 0.95 +/- 0.71 | 1.6 | 0.038 | (28/38) | 74 |
| SNX12 | sorting nexin 12 | 0.498 +/- 0.35 | 0.181 +/- 0.2 | 2.8 | 0.037 | (32/38) | 84 |
| HADHB | hydroxyacyl-CoA dehydrogenase/3-ketoacyl-CoA thiolase/enoyl-CoA hydratase (trifunctional protein), beta subunit | 2.43 +/- 1.1 | 1.65 +/- 1.1 | 1.5 | 0.041 | (27/38) | 71 |
| APOA4 | apolipoprotein A-IV | 2.18 +/- 1.2 | 3.07 +/- 1.5 | 0.7 | 0.041 | (10/38) | 26 |
| ATP6V1A | ATPase, H+ transporting, lysosomal 70kDa, V1 subunit A | 2.33 +/- 1.2 | 1.57 +/- 1.1 | 1.5 | 0.041 | (25/38) | 66 |
| AKR1B1 | aldo-keto reductase family 1, member B1 (aldose reductase) | 1.75 +/- 0.85 | 1.1 +/- 1 | 1.6 | 0.04 | (28/38) | 74 |
| ANXA6 | annexin A6 | 5.58 +/- 3.6 | 7.51 +/- 3.3 | 0.7 | 0.042 | (14/38) | 37 |
| HSPA1B | heat shock 70kDa protein 1B | 6.73 +/- 2.7 | 5.38 +/- 2.6 | 1.2 | 0.041 | (25/38) | 66 |
| RPS3A | ribosomal protein S3A | 2.82 +/- 0.93 | 1.98 +/- 1 | 1.4 | 0.042 | (30/38) | 79 |
| CNN3 | calponin 3, acidic | 1.92 +/- 1.1 | 1.24 +/- 0.82 | 1.6 | 0.042 | (24/38) | 63 |
| NNMT | nicotinamide N-methyltransferase | 0.839 +/- 0.58 | 0.413 +/- 0.34 | 2 | 0.042 | (28/38) | 74 |
| KTN1 | kinectin 1 (kinesin receptor) | 2.7 +/- 1.8 | 1.88 +/- 1.2 | 1.4 | 0.043 | (23/38) | 61 |
| ACAT1 | acetyl-CoA acetyltransferase 1 | 2.4 +/- 1.5 | 1.64 +/- 0.8 | 1.5 | 0.044 | (21/38) | 55 |
| CAPZB | capping protein (actin filament) muscle Z-line, beta | 2.19 +/- 1 | 1.47 +/- 0.75 | 1.5 | 0.043 | (26/38) | 68 |
| PRPF19 | pre-mRNA processing factor 19 | 1.23 +/- 0.59 | 0.705 +/- 0.46 | 1.8 | 0.043 | (28/38) | 74 |
| RPL9 | ribosomal protein L9 | 2.25 +/- 0.91 | 1.52 +/- 0.98 | 1.5 | 0.043 | (25/38) | 66 |
| SPR | sepiapterin reductase (7,8-dihydrobiopterin:NADP+ oxidoreductase) | 1.15 +/- 0.59 | 0.641 +/- 0.48 | 1.8 | 0.044 | (26/38) | 68 |
| HINT1 | histidine triad nucleotide binding protein 1 | 0.945 +/- 0.54 | 0.493 +/- 0.48 | 1.9 | 0.044 | (27/38) | 71 |
| DCXR | dicarbonyl/L-xylulose reductase | 0.792 +/- 0.57 | 0.386 +/- 0.42 | 2 | 0.044 | (29/38) | 76 |
| GOT2 | glutamic-oxaloacetic transaminase 2, mitochondrial | 1.78 +/- 0.71 | 1.14 +/- 0.6 | 1.6 | 0.048 | (29/38) | 76 |
| GSR | glutathione reductase | 1.34 +/- 0.78 | 0.796 +/- 0.47 | 1.7 | 0.047 | (25/38) | 66 |
| HEBP2 | heme binding protein 2 | 0.555 +/- 0.32 | 0.227 +/- 0.15 | 2.4 | 0.046 | (31/38) | 82 |
| UBE2L3 | ubiquitin-conjugating enzyme E2L 3 | 1.18 +/- 0.57 | 0.669 +/- 0.37 | 1.8 | 0.046 | (28/38) | 74 |
| VCP | valosin containing protein | 6.74 +/- 1.8 | 5.45 +/- 1.6 | 1.2 | 0.048 | (25/38) | 66 |
| TUBA1B | tubulin, alpha 1b | 4.56 +/- 2.4 | 5.77 +/- 3.2 | 0.8 | 0.048 | (14/38) | 37 |
| AP1B1 | adaptor-related protein complex 1, beta 1 subunit | 2.32 +/- 0.82 | 1.59 +/- 0.79 | 1.5 | 0.048 | (29/38) | 76 |
| EIF2S1 | eukaryotic translation initiation factor 2, subunit 1 alpha, 35kDa | 1.31 +/- 0.47 | 0.778 +/- 0.48 | 1.7 | 0.048 | (28/38) | 74 |
| YWHAG | tyrosine 3-monooxygenase/tryptophan 5-monooxygenase activation protein, gamma | 2.78 +/- 1 | 1.97 +/- 0.94 | 1.4 | 0.048 | (25/38) | 66 |
| HNRNPF | heterogeneous nuclear ribonucleoprotein F | 1.63 +/- 0.74 | 1.03 +/- 0.6 | 1.6 | 0.048 | (28/38) | 74 |
| TMSB10 | thymosin beta 10 | 0.987 +/- 0.59 | 0.532 +/- 0.62 | 1.8 | 0.048 | (28/38) | 74 |
| DNPH1 | 2'-deoxynucleoside 5'-phosphate N-hydrolase 1 | 0.47 +/- 0.33 | 0.176 +/- 0.13 | 2.7 | 0.048 | (30/38) | 79 |
| UQCRC2 | ubiquinol-cytochrome c reductase core protein II | 1.8 +/- 1 | 1.16 +/- 0.85 | 1.5 | 0.049 | (29/38) | 76 |

^a^ Values represent covariate adjusted mean spectral counts ± StDev

^b^ Ratio of means relative to control

^c^ p-values are based on paired t-test

^d^ Ratio represents the number of tumor samples which indicated a higher abundance relative to matched control tissue

^e^ Percent (%) represents number of cases were the respective protein was increased in tumor relative to control

**Table S3.** OPLSDA Model Validation Statistics

| type | AUC | Sens | Spec |
| --- | --- | --- | --- |
| model | 0.789 -/+ 0.098 | 0.735 -/+ 0.14 | 0.842 -/+ 0.14 |
| permuted | 0.501 -/+ 0.14 | 0.493 -/+ 0.21 | 0.509 -/+ 0.22 |
| p.value | 0.0297 | 0.168 | 0.099 |
| test data | 0.75 | 0.75 | 0.75 |

**Table S4.** Associations between LASP1 and lymphovascular infiltration and overall survival.

|  | **Tumor^a^** | **Control^a^** | **Fold Change^b^** | **p-value^c^** | **Ratio^d^** | **Percent (%)^e^** |
| --- | --- | --- | --- | --- | --- | --- |
| ***LASP1*** |  |  |  |  |  |  |
| Without LV Infiltration | 1.28 ± 0.5 | 1.1 ± 0.6 | 1.16 | 0.175 | (19/32) | 59 |
| With LV Infiltration | 1.94 ± 0.8 | 1.34 ± 1.1 | 1.44 | 0.337 | (4/6) | 67 |
| Alive | 1.22 ± 0.5 | 1.22 ± 0.7 | 1 | 0.976 | (12/24) | 50 |
| **Deceased** | **1.67 ± 0.6** | **1.02 ± 0.6** | **1.65** | **0.011** | **(11/14)** | **79** |

^a^ Values represent covariate adjusted mean spectral counts ± StDev

^b^ Ratio of means relative to control

^c^ p-values are based on paired t-test

^d^ Ratio represents the number of tumor samples which indicated a higher abundance relative to matched control tissue

^e^ Percent (%) represents number of cases were the respective protein was increased in tumor relative to control

^f^ LV- lymphovascular

**Table S5.** Patient Characteristics for Oncomine Dataset

| Variable | NSCLC Adenocarcinoma Subjects |
| --- | --- |
| Total Sample Size, N | 168 |
| Gender, N (Male/Female) | 71/97 |
| Age, median (range) | 61 (34-76) |
| Smoking Status, N |  |
| Never Smoker | 94 |
| Former or Current Smoker | 74 |
| Stage |  |
| IA | 114 |
| IB | 54 |
| LASP1, median (range)* | 5.64 (4.41-7.11) |

*log_2_-median centered values

**Supplemental Figure Legends**

**Figure S1. mRNA expression of top candidates in independent dataset of early stage adenocarcinoma relative to control.** Gene expression data was obtained from Okayama Lung Study. Only Stage I adenocarcinomas were considered. Significance was determined by Mann-Whitney U-test. ***<0.0005, ****<0.0001. No significant differences were observed for *SCL4A1*, *SPTB, SPTA1* and *ANK1*. *STOML2* was not detected in the dataset.

**Figure S2**. **Immunohistochemistry Scores for EPRS, HYOU1 and LASP1 in LC003 TMA.** Immunostaining scores are shown for each matched malignant and control tissue pairs. The staining intensity was graded as 0, negative (no cells stained); 1, weak; 2, moderate; 3 strong.

**Figure S3. Receiver Operating Characteristic Curve for LASP1 in Subjects who Died from NSCLC Adenocarcinoma.** ROC Curve is shown for LASP1 expression based on proteomic findings in subjects who died as a consequence of NSCLC adenocarcinoma.

**Figure S4. Kaplan-Meier Survival Curves for *LASP1* mRNA Expression Quantiles in Oncomine Okayama Lung Cancer Study.** Subjects with missing meta-data for overall survival were excluded from the analysis.

**Supplemental Figure S1
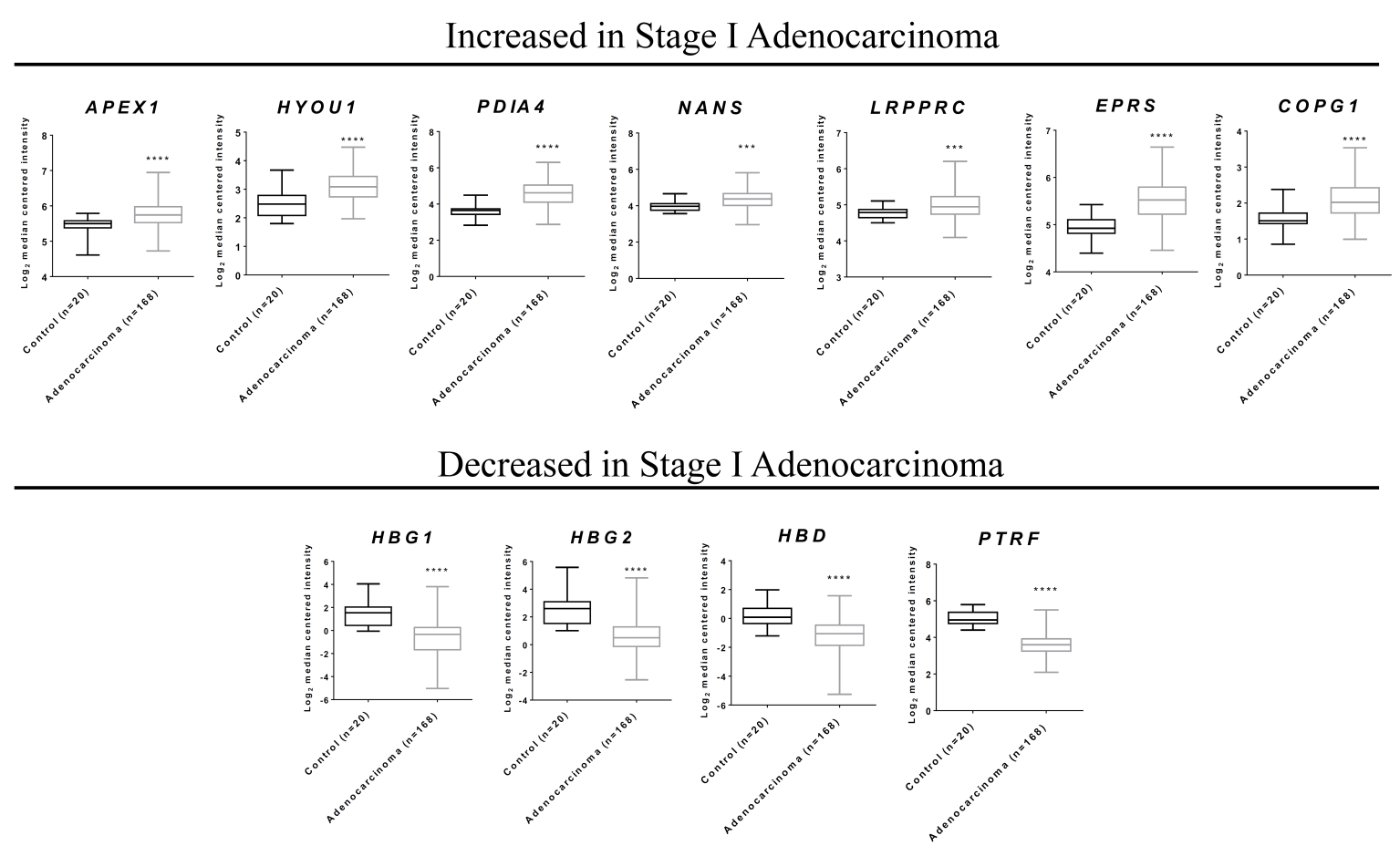
**

**Supplemental Figure S2**

**Supplemental Figure S3**

**Supplemental Figure S4**
